# Supplementary material for: Preclinical pharmacology of patient-derived extracellular vesicles for the intraoperative imaging of tumors
Source: Theranostics. 2024 Sep 30;14(16):6301–18. doi: 10.7150/thno.98671 (PMC11488097; doi:10.7150/thno.98671)
Supplement: Supplementary file 1 — Supplementary methods, figures and tables. [file thnov14p6301s1.pdf]

Preclinical pharmacology of patient-derived extracellular vesicles for the intraoperative imaging of tumors.

Alessandro Villa[1], Daniela Crescenti[1], Zemira De Mitri[1], Elisabetta Crippa[1], Silvia Rosa[1], Nicoletta Rizzi[3], Fereshteh Shojaei-Ghahrizjani[1], Monica Rebecchi[1], Simona Vincenti[2], Francesca Selmin[3], Electra Brunialti[1], Nicolò Simonotti[5], Marianna Maspero[5], Michele Dei Cas[1], Camilla Recordati[4], Saverio Paltrinieri[4], Alessia Giordano[4], Rita Paroni[1], Margherita Galassi[6], Vito Ladisa[6], Flavio Arienti[6], Francesco Cilurzo[3], Vincenzo Mazzaferro[5,6], Paolo Ciana[1]

1. Department of Health Sciences, University of Milan, Milan, Italy.
2. Department of Clinical Veterinary Medicine, Vetsuisse Faculty, University of Bern, Bern, Switzerland.
3. Department of Pharmaceutical Sciences, University of Milan, Milan, Italy
4. Department of Veterinary Medicine and Animal Sciences, University of Milan.
5. Department of Oncology and Hemato-Oncology, University of Milan, and HPB Surgery and Liver Transplantation
6. Istituto Nazionale Tumori IRCCS Foundation (INT), Milan, Italy.

Abstract

Extracellular vesicles (EVs) derived from the plasma of oncological patients exhibit significant tumor-targeting properties, unlike those from healthy individuals. We have previously shown the feasibility of formulating the near-infrared (NIR) fluorescent dye indocyanine green (ICG) with patient-derived extracellular vesicles (PDEVs) for selective delivery to neoplastic tissue. This staining protocol holds promise for clinical application in intraoperative tumor margin imaging, enabling precise neoplastic tissue resection. To this end, we propose the ONCOGREEN protocol, involving PDEV isolation, ICG loading, and reinfusion into the same patients. **Methods**. By in vivo studies on mice, we outlined key pharmacological parameters of PDEVs-ICG for intraoperative tumor imaging, PDEV biodistribution kinetics, and potential treatment-related toxicological effects. Additionally, we established a plasmapheresis-based protocol for isolating autologous PDEVs, ensuring the necessary large-scale dosage for human treatment. A potential lyophilization-based preservation method was also explored to facilitate the storage and transport of PDEVs. **Results**. The study identified the effective dose of PDEVs-ICG necessary for clear intraoperative tumor margin imaging. The biodistribution kinetics of PDEVs showed favorable targeting to neoplastic tissues, without off-target distribution. Toxicological assessments revealed no significant adverse effects associated with the treatment. The plasmapheresis-based isolation protocol successfully yielded a sufficient quantity of autologous PDEVs, and the lyophilization preservation method maintained the functional integrity of PDEVs for subsequent clinical application. **Conclusions**. Our research lays the groundwork for the direct clinical application of autologous PDEVs, initially focusing on intraoperative imaging. Utilizing autologous PDEVs has the potential to accelerate the integration of EVs as a targeted delivery tool for anti-neoplastic agents to cancerous tissue. This approach promises to enhance the precision of neoplastic tissue resection and improve overall surgical outcomes for oncological patients.

Keywords: EV Biodistribution Kinetics, Toxicology, Intraoperative Imaging, Bench-to-bedside Translation.

Email addresses: Villa A.: alessandromaria.villa@unimi.it; Crescenti D.: daniela.crescenti@unimi.it, De Mitri Z.: zemira.demitri@unimi.it; Crippa E.: elisabetta.crippa@unimi.it; Rosa S.: silvia.rosa4@studenti.unimi.it; Rizzi N.: nicoletta.rizzi@unimi.it; Shojaei-Ghahrizjani F. : fereshteh.shojaeighahrizjani@unimi.it; Rebecchi, M. : monica.rebecchi@unimi.it; Vincenti S.: simona.vincenti@unibe.ch; Selmin F. : francesca.selmin@unimi.it; Brunialti E.: electra.brunialti@unimi.it; Simonotti N.: nicksimonotti95@gmail.com; Maspero M.: mariannamaspero@gmail.com; Recordati C. : camilla.recordati@unimi.it; Paltrinieri S. : saverio.paltrinieri@unimi.it; Giordano A.: alessia.giordano@unimi.it; Galassi M.: margherita.galassi@istitutotumori.mi.it; Ladisa V. : vito.ladisa@unimi.it; Arienti F. : flavio.arienti@istitutotumori.mi.it; Cilurzo F. : francesco.cilurzo@unimi.it; Mazzaferro V. : vincenzo.mazzaferro@istitutotumori.mi.it; Ciana P. : paolo.ciana@unimi.it

Introduction

The necessity of precision medicine in oncology is becoming increasingly evident, emphasizing the critical need of precise drug delivery systems. These systems are designed to significantly enhance the effectiveness of current diagnostic and therapeutic approaches by ensuring that drugs are delivered directly to cancer cells with high specificity. Such targeted delivery would not only improve treatment efficacy but also minimize adverse side effects, thereby enhancing patient outcomes. Additionally, this precision in drug delivery would benefit from a more tailored approach to treatment, addressing the unique genetic and molecular profile of each patient's tumor. In this context, recent research has focused on the development of diverse drug delivery systems for cancer therapies, emphasizing the enhancement of the effectiveness of current therapies [1]. Polymer-based drug delivery systems have shown promise in improving the pharmacokinetics and therapeutic potential of chemotherapy [2]. Aptamer-based smart targeting and spatial trigger-response drug delivery systems have been explored for achieving precise and targeted drug delivery in anticancer therapy [3]. Furthermore, various targeting ligands, such as folic acid, carbohydrates, peptides, aptamers, antibodies, and membrane proteins have been investigated for their potential in targeted drug delivery systems for tumor therapy [4]. In this field, biomimetic solutions such as cell membrane-based nanoparticles have been developed and investigated as a new platform for tumor diagnosis and treatment, with a focus on their ability to interact with the tumor microenvironment [5]. A subsequent evolution in the development of biomimetic nanoplatforms for cancer theranostics has been the development of stem cell membranes, stem cell-derived exosomes, and hybrid stem cell-camouflaged nanoparticles [6], which have shown promise in improving drug delivery to tumors and reducing side effects [7, 8]. Exosome membrane-coated nanosystems, in particular, have been explored for their potential in cancer diagnosis and therapy, offering enhanced biocompatibility, immune evasion, and active targeting properties [9, 10]. These nanosystems are inspired to the naturally-occurring extracellular vesicles (EVs), which are nanoparticles included in a lipid bilayer membrane secreted by cancer cells, that have shown great potential in cancer diagnosis and therapy. The main characteristics of EVs is their content in nucleic acids, proteins, and metabolites, forming an informational payload that is precisely delivered to target cells through paracrine or endocrine mechanisms [11, 12]. Consequently, circulating EVs are now recognized as a novel way of communication within multicellular organisms, contributing to various biological processes, including embryonic development, coagulation, hematological processes, and organ homeostasis [13, 14].

Interfering with this communication layer by altering EV content or employing these nanoparticles as drug delivery vehicles is regarded as an innovative therapeutic strategy for numerous diseases, including cancer [15, 16]

The growing interest in using EVs as delivery tools is rooted in their intrinsic targeting properties: it has been postulated that the cellular origin unequivocally determines the homing characteristics of these nanoparticles [17, 18]. Several studies have established that EVs derived from tumor cell lines [19-23] or mesenchymal stem cells [23, 24] exhibit selective tropism for neoplastic tissue. Notably, EVs can be loaded with various exogenous molecules, such as small chemical compounds, biological macromolecules, and even an entire virus [25]. When introduced into a cancer-bearing organism, EVs originating from mesenchymal or tumoral cells, loaded with therapeutics, can selectively deliver their cargo to the neoplastic tissue [26-28]. In this application, EVs exhibit compelling attributes: they are biocompatible nanoparticles with minimal immunogenicity and toxicity, do not accumulate due to catabolic degradation, shield cargo from metabolic processes [29], and also protect the body from off-target effects when therapeutics are systemically administered to patients [30]. At present, the main obstacles impeding their clinical adoption are the challenges linked to scaling up and standardizing EV production [31], or safety concerns related to the potential oncogenic cargo present in tumor-cell line-derived EVs [32-37]. Another significant constraint is the limited understanding of the tumor homing mechanism. Once unveiled, this would signify a milestone that could potentially pave the way for the development of semi-synthetic or entirely synthetic nanoparticles [38], solving the production standardization and safety issues, thus sidestepping the transformation potential linked to EVs of tumoral origin [32, 33, 37].

Aiming at circumventing the limitations of the currently proposed methods to transport drugs to the tumor, we proposed an alternative approach for the clinical application of EVs as delivery tools for anti-neoplastic agents, termed "AUTOTERANOST" [39, 40]. This approach is based on the observation that the plasma of oncological patients (but not healthy individuals) is enriched with EVs displaying tumor tropism [20, 26, 39]. In the protocol established by AUTOTERANOST, EVs produced by the oncological patient are isolated from the plasma, loaded with therapeutics, and prepared for subsequent re-infusion into the same patient who generated the nanoparticles. The advancement provided by this method relies on merging the precise delivery of anti-neoplastic agents with a biologically compatible formulation inherently tailored for targeting tumor tissue [27, 39, 41], without affecting the

oncogenic potential of tumor-derived EVs which were circulating in the patient. In this study, our focus is on the application of autologous EVs for the delivery of a diagnostic drug, Indocyanine Green (ICG), to label tumor margins and aids surgeons in the complete removal of neoplastic tissue. The objective of this work is to characterize key pharmacological and toxicological parameters required for the application in clinics of the AUTOTERANOST protocol, allowing intraoperative imaging of neoplastic tissue using ICG formulated with patient-derived EVs (PDEVs-ICG). Moreover, since storage is one of the main hurdles of EVs in clinical practice, a freeze-dried formulation enabling the maintenance of physicochemical and functional features over time was established.

Methods

Reagents

Reagents were purchased from Sigma-Aldrich St. Louis, MO, USA if not otherwise specified.

EV extraction from blood of colorectal cancer (CRC) patient

Venous blood (15 ml) was collected from 3 patients during preoperative analyses after approval by the Ethics Committee of the National Cancer Institute of Milan (Aut. INT 244/20). Blood was collected in EDTA-conditioned vials and immediately centrifuged at 1750 g for 10 minutes at room temperature to remove blood cells and prevent platelet activation and release of platelet-derived EVs. Supernatants were transferred to new tubes (the bottom 10% of supernatant above blood cells was discarded), and samples were centrifuged again at 3000 g for 10 minutes at room temperature. Supernatants were collected and processed by ultracentrifugation for 2 hours at 100,000 g at 4 °C in an Optima L-80 XP ultracentrifuge (Beckman Coulter) with rotor SW32Ti (Beckman Coulter). Supernatants were aspirated and the EV-containing pellets were resuspended in 100 µL phosphate-buffered saline (DPBS, EMD Millipore) and stored at -80 °C until use.

Size distribution determination by nanoparticle tracking analysis (NTA)

Size distribution and concentration of EVs, and EVs formulated with ICG were analyzed by NTA using Nanosight model LM14 (Nanosight, Malvern) equipped with blue (404 nm, 70 mV) laser and sCMOS camera. NTA was performed for each sample by recording three 90 s videos, subsequently analyzed using NTA software 3.0 (Nanosight, Malvern). The detection threshold was set to level 5 and camera to level 13.

Cryo Electron Microscopy (Cryo-EM)

Cryo-EM images (150 fields) were acquired with a JEOL electron microscope equipped with a FEI Falcon 3EC direct electron detector and Volta Phase-plate. Prior to Cryo-EV imaging, the samples were vitrified on a FEI Vitrobot IV system and processed as previously reported [39].

Immunoblotting

For immunoblotting, EVs were isolated from patients' blood according to the protocol described above. After the ultracentrifugation step, the supernatants were removed, and the EV-containing pellets were resuspended in a proper volume of 1X RIPA buffer (150mM NaCl; 1% NP-40; 0.5% sodium deoxycholate; 0.1% SDS; 50 mM Tris-HCl, pH 8.0) supplemented with protease inhibitor cocktail (Roche). EV protein concentrations were quantified using a Bradford assay kit (Thermo Scientific). Twenty micrograms of EV protein lysates were separated to 4-10% SDS-PAGE using beta-mercaptoethanol as reducing agent and transferred to nitrocellulose membranes (Amersham). The membranes were then blocked in 5% nonfat dry milk in TBS-T (0.2% Tween® 20) at RT and incubated overnight with the primary antibodies against exosomal TSG101 (4A10 Abcam, 1:500) and CD9 (C9993 Sigma, 1:500). Immunoreactive bands were visualized with chemiluminescence using the ECL Western Blotting Analysis System according to the manufacturer's instructions (Amersham).

EV loading with ICG

ICG was loaded into patient-derived EVs (PDEVs) as previously described [39]. Briefly, 1E09 PDEVs were suspended in 50 µL DPBS and were added to 150 µL of a water solution of 5 mg/mL ICG (Sigma) and incubated for 12 hours at 4 °C. Then, samples were centrifuged

at 100,000 x g for 90 min; after supernatants removal, pellets were resuspended in 150 µL of DPBS.

Quantification of ICG incorporated into EVs

Quantification of ICG was performed by both high-performance liquid chromatography (HPLC) coupled with a UV detector (LC-UV) and high-resolution liquid chromatography coupled with a mass spectrometer (LC-HRMS /MS). For quantification by HPLC, an HPLC with UV detector (mod. 1000) and autoprobing (mod. 4000) was used with a Phenomenex, Bondclone 10 µm C18, 300x3.9 as column. The flow rate was 0.8 mL/min with a UV length of 250 nm and an injection volume of 10 µL. IR-806 (Sigma-Aldrich) was used as an internal standard because its spectrochromatographic molecular properties are similar to those of ICG. For the analysis, a solution of 1 mg/mL IR-806 in ethanol was prepared. This solution was then diluted with water to give a concentration of 25 ug/ml. The ICG (Verdye solution 5mg/mL) was titrated at a concentration of 5 mg/mL. To establish the calibration line, an initial dilution was made from the ICG stock to obtain a final concentration of 1 mg/mL ICG. This was followed by a 1:100 dilution and finally serial 1:2 dilutions. Vials for the calibration line were prepared by adding 10 µL internal standard (25 µg/mL) to 10 µL standard solutions and 180 µL water, resulting in a linearity range of 50 µg/mL to 0.163 µg/mL ICG. Quantification of samples was performed using the Shimadzu UPLC instrument coupled to the Triple TOF 6600 Sciex (Concord, ON, CA) equipped with the Turbo Spray IonDrive. All samples were analyzed by electrospray ionization (ESI) in positive polarity (a mild ionization technique usually used for substances that are in solution in ionic form or are readily ionized). The analytical conditions are as follows: GS1 (nebulizer gas): 55, GS2 (drying gas): 65, CUR: 35, with a capillary voltage: 5.5kV, a temperature of 500 °C at the source, 45 °C in the column, a dusting potential of 70 eV, an ionization energy: 70 ± 15 eV. The column was a reversed phase Acquity HSS T3 C18 column 1.7 µm, 2.1100 mm (Waters, Franklin, MA, USA) equipped with a precolumn; the mobile phase: (A) water and (B) acetonitrile. Both contained 0.1% formic acid and a flow rate: 0.4 mL/min. Under these conditions, ICG has a retention time of 3.2 minutes and peaks at 821.9 m/z. Subsequent fragmentation of ICG results in a higher peak at 374.98 m/z, which is used for quantification.

Animals

All the animal experiments were performed and approved by the Italian Ministry of Research and University (permission number: 214/2020) and regulated by a departmental panel of experts. C57BL/6NCrl (Charles River, MGI: 2683688) mice were maintained at the animal facility of the University of Milan under standard conditions according to institutional guidelines. After an acclimatization period of 14 days, murine syngeneic grafts were established by s.c. injections of 2E06 MC-38 cells into the neck of 12-week-old male C57BL/6 mice. The health status of mice in the experimentation was monitored daily, and as soon as signs of pain or distress were evident, the mice were euthanized. The size of the tumor was measured using a caliper, using the calculation formula V= 1/2 ×length×(width)^2, where: V is the tumor volume, the length is the longest diameter of the tumor, and the width is the shortest diameter perpendicular to the length. For the homing tests, mice engrafted with tumors were i.v. injected with different doses of PDEVs-ICG

In vivo and ex vivo fluorescence imaging

In vivo and *ex vivo* fluorescence imaging sessions were carried out 24 h after EV treatment using the preclinical imaging instrument IVIS Spectrum (Perkin-Elmer) and the SPY Elite intraoperative imaging device (Stryker, USA), equipped with filters for NIR signal detection, following the manufacturer instructions. Mice were anaesthetized using isoflurane (Isoflurane-V, Merial, Lyon, France) and kept under anesthesia during imaging sessions carried out with the imaging system. For *ex vivo* imaging, mice were sacrificed by cervical dislocation. Immediately after death, selected organ imaging was also carried out.

Hematology and clinical chemistry

Blood (approximately 500 μL) was collected in heparinized tubes and stored at 4°C until analysis. Routine hematology was performed using a laser-based cell counter (Sysmex XN-V, Sysmex Co. Kobe, Japan), followed by microscopical analysis of May Grünwald-Giemsa stained smears to check the accuracy of the automated differential cell count. After the cell count, blood samples were centrifuged, and plasma was harvested to perform biochemical analysis. Clinical chemistry was performed using an automated spectrophotometer (BT3500, Biotecnica Instruments SPA, Roma, Italy) and measuring the following analytes using reagents provided by Futurlab S.r.l. (Limena, PD, Italy): cholesterol, glucose, total protein, albumin, creatinine, urea, alanine aminotransferase (ALT), glutamate

dehydrogenase (GLDH), alkaline phosphatase (ALP), creatine kinase (CK) and lactate dehydrogenase (LDH). Citrated plasma was used on an optical coagulometer (Coatron M1 TECO GmbH, Germany) to measure fibrinogen by converting the clotting time under bovine thrombin activation, the prothrombin time (PT) after the addition to plasma of thromboplastin and calcium chloride and the activated partial thromboplastin time (aPTT) after the addition to plasma of ellagic acid, phospholipids and calcium chloride.

Bone marrow cytology

After sacrifice and before to perform a complete necropsy, bone marrow was collected from the femur using a 24G syringe needle, smeared on glass slides and air dried. May Grünwald-Giemsa stained smear were microscopically analyzed to count at least 500 nucleated cells: the following indicators were recorded: megakaryocyte number and morphology, myeloid:erythroid (M:E) ratio; percentage of cells belonging to the proliferative pool (P, composed by blasts able to divide) and to the maturation (M) pool, followed by the calculation of the P:M ratio, either for the erythroid lineage or for the myeloid lineage; percentage of lymphocytes.

Lyophilization process

Compatibility tests between EVs and cryoprotectant-lyoprotectant solutions were conducted. Compatibility was assessed by evaluating changes in particle size and number before and after the addition of the excipient using NTA. EV dispersions in 10x PBS were frozen and stored in a freezer at -80 °C until use. Upon thawing, trehalose solutions or a mixture of trehalose and sucrose in a 50:50 ratio, previously filtered through a 0.22 µm nominal pore size membrane (VWR International, Italy), were added. The resulting dispersions were evaluated for stability following freezing and thawing. The EV dispersion in the presence of cryo-/lyo-protectants underwent a freezing and thawing process to better understand the effect of ice crystal formation, as well as the effect of the initial phase of the lyophilization process. The freezing and thawing process was conducted in the furnace of a DSC Mettler StarE (Mettler Toledo – Switzerland). Approximately 30 µL of accurately weighed sample in a 40 µL aluminum crucible was subjected to the following thermal cycle in a nitrogen atmosphere with a flow rate of 80 mL/min: ramp at 2 K/min from 25 to -45 °C; isothermal at -45 °C for 5 min; ramp at 1 K/min from -45 °C to 10 °C. For lyophilization, glass vials of type R2 (Schott - Germany) were filled with 0.3 mL aliquots of the different

formulations and excipient solution to achieve a volume of 280 mL. Subsequently, the vials were partially closed with rubber stoppers and placed in the central tray of the freeze dryer (Epsilon 2-6D LSCplus, Martin Christ - Germany) in a hexagonal arrangement to maximize heat conduction. The temperature of the samples was monitored and recorded by 3 wired probes and 2 wireless probes placed in the vials at the central position. Based on the results of the freezing and thawing tests, EVs in the presence of trehalose or trehalose-sucrose mixtures in ratios of 30:70, 50:50, and 70:30 were subjected to the lyophilization process with the following parameters: *Step 1*. Freezing Rate (K/min) 1.5; Pressure (mBar) 1000; Temperature (°C) -40 °C; holding time (h) 8. *Step 2*: Freezing Rate (K/min) --; Pressure (mBar) 0.100; Temperature (°C) -40; holding time (h) 22. Step 3: Freezing Rate (K/min) 0.1; Pressure (mBar) 0.1; Temperature (°C) 20; holding time (h) 4.

Results

Intraoperative detection of tumor margins by PDEVs: dose finding in mice

Our previous experimentations carried out in animal models, including mice and dogs, indicated the possibility to administer the PDEVs-ICG formulation for the specific labeling of neoplastic tissue with the fluorescent dye [20, 26, 39]. This specificity in tumor targeting, which cannot be observed for EVs derived from the plasma of healthy subject [39], refers to their unique capability to target tumoral tissue versus healthy tissue, irrespective of the specific tumor under study. To optimize this protocol for intraoperative imaging of tumor margins in humans, we initially conducted experiments to define the optimal dosage of ICG formulated with the PDEVs that is required to deliver a sufficient quantity of the NIR dye allowing the detection of the fluorescent radiation. For these experiments, we used PDEVs isolated from the plasma of two patients diagnosed with CRC (Aut. INT 244/20) which were characterized in accordance with the recommendations outlined in the document titled 'Minimal Information for Studies of Extracellular Vesicles (MISEV2023)' [42]; the nanometric characteristics of the isolated PDEVs are reported in Table 1, while the complete characterization of these EVs was reported previously [39].

Furthermore, we evaluated the efficacy of our loading protocol by quantifying the ICG content in the PDEVs-ICG formulations. Referred to as the ONCOGREEN formulation, the process involves passive incubation of PDEVs suspended in DPBS with a 5 mg/mL ICG solution at 4°C for 12 hours. Subsequently, the PDEVs underwent ultracentrifugation to remove excess dye, and the amount of dye integrated into the PDEVs was determined through LC-MS analysis. On average, the passive loading procedure efficiently incorporated a total of 150 nmol of ICG (0,116 mg) into 1E08 PDEVs (Supplementary Figure 1).

Following the measurement of the quantity of ICG incorporated into our ONCOGREEN preparation, we set out to determine the Minimum Effective Dose (MED) required for detecting tumor margins with intraoperative instrumentation. To this purpose, we used a well-established *in vivo* method routinely used in our laboratory to investigate the tropism capabilities of PDEVs. This method involves intravenously administering the EVs to mice with tumors, followed by assessing the biodistribution of these fluorescent nanoparticles 24 hours post-injection [39]. In this experiment, three distinct dosages of ONCOGREEN were evaluated across three experimental groups. Each group included eight C57BL/6 wild-type

mice, totaling 24 mice, that were subcutaneously implanted with the syngeneic MC-38 colorectal cancer cell line in the neck area (supplementary Figure 2). Thirteen days post-implantation of cancer cells, when the tumor volume reached approximately 300-400 mm^3, the animals received a single intravenous dose of ONCOGREEN. The dosage varied based on the experimental group: 3.3E07 EVs/Kg, 3.3E08 EVs/Kg, and 3.3E09 EVs/Kg, respectively (Figure 1A).

Twenty-four hours after the treatment, the mice were subjected to *in vivo* imaging sessions to detect the fluorescent signal released by the PDEVs. The optimal time frame of 24 hours for *in vivo* ICG detection was determined during our previous experiments [20, 26, 39, 43, 44]. In these studies, we also examined the *in vivo* biodistribution of PDEVs-ICG over extended periods, showing that the fluorescence detected in the tumor area is suitable for intraoperative imaging at 24 hours and begins to decline by 48 hours. At 24 hours, we observed that mice injected with the highest dose of ONCOGREEN emanated a distinct fluorescent signal from the neoplastic tissue, a result that was consistent with the findings of our earlier researches [20, 39] (Fig. 1B and supplementary Figure 3). In this experimental group, the fluorescent signal coming from the tumor area was clearly visible, even when using intraoperative imaging instrumentation (the Stryker SPY Elite instrument) to detect the fluorescence emission. It's noteworthy that this highest dosage contained approximately 4 mg/Kg of ICG, which is lower than the approved human dose (5 mg/Kg), and 100 times lower than the LD50 [45, 46]. Conversely, the signal was hardly visible in the group of mice treated with a dose of 3.3E08 EVs/Kg, and undetectable at the lowest dose, suggesting that the MED for detecting tumor margins with intraoperative instrumentation was 3.3E09 EVs/Kg.

Subsequently, we conducted additional experiments to investigate the influence of tumor size on the intensity of fluorescence detected by the intraoperative imaging system. We divided mice into three groups, each consisting of three mice, totaling nine mice. They were categorized based on tumor size as small (0.10-0.25 cm^3), medium (0.30-0.55 cm^3), and large (0.70-1.00 cm^3).

Twenty-four hours after the treatment, they were euthanized for direct *ex vivo* measurement of the fluorescence emitted by the tumors. The quantification of the fluorescent emission was divided by the considered detection area. Remarkably, a consistent level of photon emission was observed across all tumor sizes, suggesting a uniform uptake of PDEVs by the tumors (Figure 2 and supplementary Figure 4). Based on this evidence, we can conclude

that under saturating conditions, the absolute intensity of fluorescent emission primarily relies on the number of neoplastic cells within the tumor (i.e., the tumor size). Further experiments were conducted to assess if the fluorescent PDEVs were able to accumulate and evidence with the fluorescent signal also very small tumors (<0.10 cm^3) originated by the subcutaneous injection of MC-38 cells. To this purpose, n=3 mice were intravenously treated with 3.3E09 EVs/Kg of the ONCOGREEN formulation and twenty-four hours after the treatment, they were subjected to *in vivo* and *ex vivo* imaging (Supplementary Figure 5). In this case, it was not possible to visualize the tumor through *in vivo* imaging due to the skin shielding effect, but the tumor tissue was marked again, as determined by *ex vivo* imaging, thus suggesting that also very small tumors in the early phase could be marked using PDEVs for intraoperative imaging.

Kinetics of biodistribution and toxicological analysis.

With the MED established at 3.3E09 PDEVs-ICG/Kg, we proceeded at characterizing the kinetics of NIR fluorescence biodistribution in murine models bearing MC-38 tumors with a volume of approximately 0.4 cm^3. In this experiment the MED of ONCOGREEN formulation or an equivalent dose of the standard formulation of free ICG were administered, and the fluorescence biodistribution was assessed at three time points (2, 24, and 96 hours) across three experimental groups, through *ex vivo* imaging on explanted organs. Two hours post-administration, we observed fluorescence peaks in the liver and kidneys, organs implicated in the excretion pathways of both free ICG and the excipient (PDEVs) (Figures 3B and 3C).

Additionally, in both treatment groups, a faint signal was observed in the lungs (Supplementary Figure 6), where previous reports have indicated some ICG accumulation [39]. These data suggest that the comparable fluorescence biodistribution observed 2 hours after administration for animals treated with free ICG and PDEVs-ICG could be attributed to the shared excretion pathway for PDEVs [45] and ICG [46, 47], and/or to the rapid metabolization of EVs immediately following injection, leading to the release of their ICG content into the bloodstream [48]. Importantly, after 24 hours, fluorescence accumulation was exclusively observed at the tumor site, reaching a peak of fluorescence that returned to baseline levels by 96 hours (Figure 3A). In conclusion, these data indicate that the optimal time frame for achieving a peak of ICG accumulation within neoplastic tissue after the administration of the ONCOGREEN formulation is 24 hours, as suggested in previous

experimental settings in canine patients [39]. Therefore, a time point of 24 hours following the infusion of ONCOGREEN should be considered optimal for intraoperative imaging of tumor margins. The subsequent step to advance the clinical application of the ONCOGREEN protocol was the toxicology assessment of the EV treatment. Safety is indeed a critical aspect for the potential translation of the protocol of autologous PDEVs infusion in humans. For this purpose, we investigated the effect of EV injection in mice key blood biochemical values and the production of pathological biomarkers related to liver function and inflammation, compared to a physiological condition in which the animals were treated with the vehicle alone. In this case, since the toxicological investigation aimed to identify any toxic effects of autologous extracellular vesicle injection, the treatments were conducted using EVs produced from primary murine fibroblast cells, syngeneic with the recipient animals, while the vehicle was DPBS, the buffer in which the EVs were suspended. We intravenously treated groups of n=8 C57Bl/6 mice with two different doses: 3.3E09 and 3.3E10 (syngeneic) EVs/Kg. Then, these groups were sacrificed at three time points: 1 hour, 24 hours, and 10 days after treatment. At the 1-hour mark, we examined coagulation parameters and bone marrow cytology (Table 2).

For the coagulation parameters, comparing results among groups was challenging due to several samples in almost all groups exhibiting coagulation times longer than the measurable scale or exceptionally high, often linked to very low fibrinogen concentrations and/or potential preanalytical factors or dilutional effects. This could be partly attributed to some samples being highly hemolytic, yet the high frequency of these occurrences suggests a potential preanalytical activation of hemostasis that may have transpired *in vitro* (e.g., during sampling or coagulation within tubes) or *in vivo*. The latter hypothesis was discarded based on histology results that reported the absence of thrombosis 1h after the treatment (Supplementary Table 1), while at 24h thrombosis was focally observed in 1 control (in the lung) and 1 treated (in the injection site and liver) mouse, suggesting a procedure-related effect (likely due to the retro-orbital i.v. injection) rather than an effect related to the treatment. No findings of thrombosis were identified 10 days after treatment. Moreover, this phenomenon seems to have manifested in both treated mice and controls, however not evidently associated with treatments. Local treatment-related effects were observed only in the high dose group, consistent with a retro-orbital macrophagic infiltration in the injection site 24h after treatment, undergoing recovery 10 days after treatment (Supplementary Table 1, and Supplementary Figure 9). Macrophagic infiltration was likely aimed at removing extravasated EVs at the level of the injection site. Bone marrow analysis did not reveal

biologically significant changes potentially associated with the treatments, except for a suspected increase in leukopoiesis at 1 hour, which was not confirmed at 24 hours and 10 days (Supplementary Figure 7). At 24 hours and 10 days after the treatment with EVs, a comprehensive evaluation was performed, assessing clinical chemistry. The study evaluated renal functions, protein profiles, enzymes, and energy metabolism following treatments (Table 3).

Glucose levels decreased slightly at both time points, but showed no dose-dependent changes, which was also observed for urea levels. Creatinine levels were slightly elevated when compared to controls in both treatment groups at 24 hours, but not at 10 days. Total protein levels showed minor variations, and albumin levels were consistent with reference intervals. Enzyme activity related to liver damage (ALT and GLDH) showed a dose-dependent increase at 24h, but significance was not reached for ALT. Conversely, GLDH showed significant differences between treatment groups, suggesting a possible liver damage in individual mice of the 3.3E10 EVs/Kg group; nevertheless, histological verification did not detect any liver damage in the treated animals (Supplementary Table 1), thus suggesting that the GLDH data may be linked to the sampling procedure. ALP activity increased without statistical significance. Muscle enzyme (CK and LDH) activity was elevated in all groups, possibly associated to a certain degree of hemolysis of samples, independently of treatment groups or time points. Overall, there were no significant dose- or time-dependent changes in treatments, with minor variations observed. GLDH showed significant differences between treatment groups, suggesting potential liver damage in individual mice of the 3.3E09 group; nevertheless, histological verification did not detect any liver damage in the treated animals (Supplementary Table 1), thus suggesting that the GLDH data could be artifacts. Also, routine hematology on peripheral blood (Table 4) did not evidence substantial changes related to the treatments.

Erythroid parameters, including RBC counts, hemoglobin, and hematocrit showed only a slight and non-significant increase in treatment groups compared to controls. Platelet counts were consistently low across all groups except for two mice (Supplementary figure 8), at all times, indicating thrombocytopenia, and may be explained with a decreased platelet production by the bone marrow, (not supported by bone marrow analysis, which conversely displays a trend to the activation of megakaryopoiesis evidenced as megakaryocytic hyperplasia (Supplementary Figure 7) in a few individual mice across all groups and time points), or – more likely - by an increased platelet consumption due to activation of coagulation during sampling or a pre-existing condition of the mice used in the

experimentation. Leukocyte parameters showed no significant changes at 24 hours post-treatment. In the 10-day experiment, the total leukocyte counts of the control group exhibited high dispersion around the median level, partly due to the presence of outliers with unusually high values. Interestingly, this dispersion resulted in a median level higher than that of the control group in the 24-hour experiment. Consequently, this variability in the control group may explain the significant difference observed with the treatment groups. Anyway, the median values of the treatment groups were quantitatively similar to those recorded in the treatment groups of the 24-hour experiment, suggesting consistency in the hematological response across different time points. Overall, the hematological analysis indicated no significant alterations in erythroid parameters and some differences in the myeloid parameters not related to the treatment. Despite some individual variations observed in blood leukocyte populations across all groups and time points, the treatments appear to have no discernible influence on blood cell populations. Notably, the observed changes were consistently present at various levels (including coagulation times, platelet count in blood, and megakaryocyte hyperplasia in bone marrow) across all groups, including controls. Overall, the analyses did not reveal any toxic effect associated with the treatment, therefore a single treatment up to 10 times the established MED can be considered safe in mice. When considering human treatment, the observed changes in hemostasis, although not supported by histopathological analysis, may suggest that attention should be given to the administration parameters, such as volume and duration, to minimize potential coagulation issues. Nevertheless, these findings are in line with numerous prior toxicological studies conducted on both autologous and heterologous EVs in rodents [20, 28, 39, 49] and with Phase 1/2 studies in humans [50, 51]. When ICG was encapsulated in EVs, the biodistribution data (Figure 3) did not reveal any new accumulation sites compared to free ICG, except within the tumor mass. This suggests that no additional side effects are expected from the ICG formulation with PDEVs compared to the standard solution. Overall, these results offer strong evidence that the delivery of ICG through PDEVs outlined in the ONCOGREEN protocol demonstrates a favorable safety profile.

Characterization of plasmapheresis-derived PDEVs

The established MED, determined to be both safe and adequate for delivering a sufficient quantity of ICG for intraoperative imaging of tumor margins, was found to be 3.3E09 PDEVs-ICG per kilogram. For example, for a patient weighing 70 kilograms, the recommended MED would be a total of 2.3E11 PDEVs-ICG. Given that the yield of PDEVs obtained from a

standard blood draw, equivalent to 4-5 mL of plasma, is approximately 3E9, it follows that the amount of plasma required to isolate such a number of EVs would be about 500 mL. To obtain this volume of plasma from a single CRC patient, plasmapheresis is a viable option. Plasmapheresis is a medical procedure allowing the separation of large volumes of blood plasma from other blood components, replacing it with a physiological solution. It is clinically acceptable in oncology patients and doesn't interfere with standard clinical procedures: for this study, plasmapheresis was authorized by the hospital's ethical committee (Aut. INT 244/20). For scaling up the procedure to handle this substantial volume of plasma safely, we set-up a clinical protocol for isolating PDEVs via plasmapheresis and manufacturing the ONCOGREEN formulation under the Good Compounding Practice of Italian Pharmacopeia in the framework of the European Pharmacopeia monograph “Pharmaceutical preparations”. This protocol has been demonstrated feasible in sterile conditions with multiple operators through a Mediafill test. The PDEVs obtained through this protocol were thoroughly characterized following the MISEV2023 recommendations [42]. In this first phase, PDEVs isolated from four CRC patients, each donating 500 ml of plasma via plasmapheresis, were tested. NTA was employed to profile their dimensions and quantify the total number. The profile analyses of PDEVs isolated following the plasmapheresis procedure reported values for dimension parameters such as D_{10}, D_{50}, D_{90}, mode, mean, as well as the total number of nanoparticles that could be obtained from these patients (Table 5 and Figure 4A), allowing the comparison with PDEVs isolated from a standard blood draw (Table 1).

Notably, the analysis reported similar dimensions for EVs isolated from plasmapheresis preparations compared to those obtained from blood draws. Moreover, the total number of nanoparticles derived from both procedures was similar (when normalized to the blood volume), being in the range of 2-9E10 EV/ml of blood. Further characterization of the EVs included the examination of the expression of the exosome biomarker Tumor Susceptibility Gene-101 (TSG101, Figure 4B), the cryo-EM imaging that verified the correct morphology and the FACS analysis of carboxyfluorescein succinimidyl ester (CFSE)-stained PDEVs to test the integrity (Figure 4C and 4D).

The CFSE stain is commonly used to assess the integrity of EVs because it can permeate the EV membrane. When CFSE enters the EVs, it reacts with intravesicular proteins, resulting in fluorescence. If the EV membrane is intact, the CFSE remains encapsulated, leading to stable fluorescence. Conversely, if the EV membrane is compromised, the CFSE leaks out, causing a strong decrease in fluorescence intensity. Therefore, by measuring the

fluorescence intensity of CFSE-stained EVs, it is possible to determine their structural integrity. This analysis demonstrated that at least 80% of nanoparticles retained their integrity and correctly incorporated the fluorescent dye (Figure 4D). Most importantly, the procedure of plasmapheresis used for the isolation of PDEVs did not impact the tumor-targeting ability of the nanoparticles, as shown by *in vivo* experiments, which confirmed consistent tumor-targeting capability (Supplementary Figure 10). In conclusion, the extensive characterization of plasmapheresis-derived PDEVs revealed dimensions, biomarker expression, morphological and functional integrity which are comparable with PDEVs isolated from standard blood draws. Moreover, PDEVs isolated with the plasmapheresis protocol retain the tumor-targeting capabilities of their counterparts isolated with the standard protocol. These findings established a solid foundation for the potential application of these PDEVs as carriers for intraoperative imaging agents, supporting the translation of this approach to human clinical use.

Lyophilized EVs retain their tumor targeting properties

While the protocol for EV isolation, loading with ICG to generate the ONCOGREEN formulation, and re-infusion in the same patient can be carried out within the hospitalization timeframe in a clinical setting, future clinical applications of PDEVs might necessitate that EVs are isolated at an earlier time, perhaps several months before their reinfusion into the patient. This approach requires the identification of suitable stabilization/storage methods that preserve at least the homing capabilities of the PDEVs. For this reason, it was examined whether lyophilization could be proposed to maintain the tumor-targeting ability of EVs. In pursuit of this goal, we set-up and applied a lyophilization procedure (detailed in the Materials and Methods section) to EVs derived from a tumor cell line (MCF-7). Briefly, these EVs were either resuspended in DPBS or subjected to lyophilization in a buffer made of a 10x PBS at pH 7.4 in a volumetric ratio of 1:1 with a 17.6% (w/v) trehalose solution. This combination of excipients was selected not only because the osmolarity of 270 mOsm/Kg complies the values required for intravenous injections, but also because it resulted suitable to stabilize EVs upon freeze-thawing since no loss on EV number and/or variation in size and size distribution was detected by NTA (Supplementary Figure 11). Upon reconstitution of the freeze-dried cake, the lyophilized EVs were divided into two sets stored at room temperature and 4 °C for 4 months, respectively. Afterwards, the lyophilized EVs were reconstituted in water and characterized using NTA and western blotting analyses. The

results revealed a comparable NTA profile in terms of dimension, distribution, and EV count with the non-lyophilized EVs (Figure 5A).

Nevertheless, a downregulation in certain protein components was also noticed, most notably for α-tubulin. This is possibly due to the fact that some proteins have a higher susceptibility to degradation induced by the lyophilization process compared to others (a comparison between α-tubulin and TSG101 in the western blot exemplifies this difference, Figure 5B). However, it is worth emphasizing that when the lyophilized and reconstituted EVs were in vivo tested in the murine models of subcutaneous tumors previously described, the biodistribution studies at 24 hours clearly demonstrated the retention of the tumor-targeting property in lyophilized EVs (Figure 5C). These results indicate that, while some alterations did occur due to after lyophilization, the functional aspect of EVs remained largely akin to the pre-lyophilization state. The lyophilization therefore can be considered as a potential preserving modality for EVs used as tools for the tumor-selective delivery of diagnostics or therapeutics, thus expanding the potential scope of clinical applications for autologous PDEVs.

Discussion

In recent years, various drug delivery strategies have been developed with the aim of delivering diagnostic and therapeutic molecules with high precision to tumor tissue. These strategies are designed to enhance the detection of tumors and reduce the incidence of off-target side effects on healthy tissues. Liposomes offer a reliable method for producing nanocarriers that protect drugs from dilution, degradation, or inactivation. However, they face biological barriers such as triggering the innate immune response, off-target accumulation, and rapid clearance from the bloodstream [52]. While some studies show effective targeting mainly due to the EPR effect, others report high non-specific accumulation in clearance organs, reducing the effective dose. Even active targeting with receptor-specific ligands or antibodies yields variable results, often suggesting that passive targeting via the EPR effect is the primary mechanism [53, 54]. The most recent cutting-edge technologies used in biomedical applications for cancer imaging and therapy, propose strategies based on biomimetic nanoparticles, such as cancer membrane-camouflaged nanoparticles [9, 10], which are engineered by cloaking their surface with cancer cell membranes to enhance their functionality in interacting with cancerous tissues. The aim of developing these biomimetic nanoparticles for cancer targeting is to achieve safe and biocompatible delivery of therapeutic agents specifically to tumor cells, enhancing treatment efficacy while minimizing

side effects [55]. In this context, PDEVs present distinct advantages over synthetic nanoparticles and biomimetic systems, particularly in the realm of biomedical applications. PDEVs are autologous, naturally sourced from the patient's own cells, circulating in bodily fluids like blood or urine. This inherent origin ensures high biocompatibility, as they are less likely to trigger immune responses or adverse reactions compared to synthetic nanoparticles [39, 56]. PDEVs also demonstrate natural tropism towards tumor tissues, possibly through surface receptors or ligands that facilitate their accumulation in cancerous sites. This innate targeting ability enhances their efficacy in delivering diagnostic or therapeutic payloads directly to tumors, potentially without requiring additional modifications or coatings that synthetic nanoparticles may need. This natural tropism observed in PDEVs is a feature which is not seen for EVs isolated from healthy subjects [39], thus indicating that the accumulation in the tumor is due to the active-targeting capacity of tumor-derived EVs, rather than to an EPR effect, which would be observed also for the EVs isolated from healthy donors. In contrast, cancer membrane-camouflaged nanoparticles, while engineered for enhanced targeting specificity and drug delivery, may lack the natural biomolecular complexity and origin inherent to PDEVs. While cancer membrane-camouflaged nanoparticles certainly offer advantages in targeted delivery and functionalization, PDEVs excel in biocompatibility, natural cargo diversity, innate targeting ability, clinical applicability, and versatility. These attributes position PDEVs as promising candidates for advancing personalized approaches to cancer imaging and therapy. The use of autologous PDEVs holds the potential to accelerate the clinical application of EVs as a tool for the targeted delivery of anti-neoplastic agents to cancerous tissue, offering significantly higher safety and biocompatibility due to their autologous origin compared to synthetic nanocarriers or EVs derived from cell cultures, which in turns might introduce exogenous oncogenes into the patient through their cargo [57], potentially leading to unforeseen developments in the natural history of the tumors present in the patients. From an experimental design perspective, focusing on PDEVs as carriers for delivering molecules to tumors required conducting pharmacokinetics studies using PDEVs. We have established the ONCOGREEN formulation as the foundation for translating preclinical proof-of-principle data obtained from our previous studies in mice and dogs [27, 39] into human applications. In particular, for clinical implementation of the ONCOGREEN formulation in intraoperative tumor margin imaging, several parameters needed to be determined, including quality control measures for plasma-derived autologous EVs, the optimal dosage, and safety aspects regarding potential ICG dye accumulation in unintended sites depending from the

excipient component (PDEVs), as well as potential toxic effects associated with the excipient dose. Therefore, we have initially determined the minimum effective dose of the ONCOGREEN formulation for tumor detection using intraoperative imaging instruments, taking MED as the reference dose for production, quality control and safety considerations. Concerning safety, we must consider that with MED dosage, we administer an ICG amount within the standard range for this drug (approximately 10 times less than the maximum dose indicated for tumor detection).

At this dose, we have not observed any unexpected accumulation of the dye in undesired locations. Conversely, at 24 hours, we observed the accumulation of a sufficient amount of ICG in tumor tissue for effective fluorescent detection through intraoperative instrumentation. Thus, we do not expect any undesired effects connected with the ectopically accumulated ICG in the ONCOGREEN formulation. We are confident that there will be no immune reactions linked to the administration of the MED: this expectation arises primarily from the fact that the PDEVs are derived from the same patient from whom they were isolated. Additionally, previous preclinical [39, 49] and phase 1 and 2 clinical trials involving patients who were administered autologous or heterologous EVs did not reveal any intrinsic toxicity [50, 58, 59].

Nevertheless, to corroborate our previous findings, the toxicology of EVs was investigated by injecting two doses of EVs derived from syngeneic cells into healthy animals to study the organism's response to autologous EV injection. This test was designed as a Phase I safety study on healthy volunteers, aiming to detect any toxicity or adverse drug reactions induced by EV doses that, at the highest dosage, exceeded the effective dose by an order of magnitude. Notably, even at this high dose, the test mice exhibited no signs of toxicity, as no significant effects were observed on the coagulation, complete blood cell count, bone marrow counts, or organ histopathology and biochemical markers. Based on these results, we confidently assert that administering autologous PDEVs once at the MED dosage is a safe procedure. Moreover, the feasibility of performing a plasmapheresis procedure on oncology patients within 48 hours prior to tumor surgical removal was thoroughly discussed with oncologists and transfusion specialists at the National Cancer Institute of Milan, which confirmed that pre-surgical plasmapheresis is a safe procedure included in the hospital protocol. This practice was routinely used until a few years ago to allow for autotransfusion of plasma if needed during the surgical operation. However, it was later discontinued as it was rarely found to be necessary. Nevertheless, it remains an available option in the therapeutic process and does not affect patient safety, the success rate of the surgical

intervention, or the patient's recovery chances. Therefore, its integration into the procedures does not represent a concern. It is also important to note that the autologous PDEVs required for the AUTOTERANOST protocol in patients will be derived from a maximum of 500 mL of plasma per patient. This amount represents approximately 15% of the total plasma - and therefore of the total vesicles already circulating in the patient's blood. Therefore, re-infusing the labeled vesicles is not anticipated to significantly alter the total EV count in the patient under pathophysiological conditions, even in cases of hepatic failure. To ensure the highest safety standards, the labeled PDEVs will be resuspended in a volume of physiological solution equivalent to the plasma volume previously drawn, and the infusion will be administered over a prolonged period (>1 hour).If the results obtained in mice and dogs should be validated in a clinical setting, it would represent the proof-of-concept that PDEVs could potentially be employed as carriers for different kind of drugs, including anti-neoplastic agents, to target the payload into the neoplastic tissue.

At this time, we are not yet aware of specific markers for tumor-derived EVs, which prevents us from accurately quantifying the contribution of PDEVs to the total circulating vesicle population to precisely define the amount of EVs that will target the tumor. Therefore, the minimum effective dose was determined by considering the total population of extracellular vesicles present in the plasma of oncology patients, hypothesizing that the size of the circulating population of tumoral EVs is consistent among all the enrolled patients. This consideration is primarily based on two concepts: first, in recent years, we have isolated EVs from a significant number of patients and observed that the total number of EVs is very similar across all the samples we have analyzed. This consistency leads us to believe that there is no significant variation in the proportion of vesicles within the different populations. Second, the proposed clinical protocol AUTOTERANOST involves the use of autologous PDEVs in patients eligible for curative surgical intervention, who are all at a similar stage of tumor development. However, we believe that tumor staging should not substantially affect the enrichment of the total circulating EV population with tumor-derived EVs. Indeed, tumoral EVs are recognized for their ability to prolong the circulation time of their therapeutic cargo, likely due to their “immunologically privileged” status. Unlike ‘physiological’ EVs and artificial nanoparticles, which are rapidly cleared by macrophages in the liver and spleen, tumoral EVs express signals that act as “do not eat me” markers, reducing phagocytic uptake [60, 61]. This suggests that vesicles continuously released by tumor cells would quickly accumulate within the circulatory system from the onset of the tumor mass, thus forming – in a short time span - the predominant circulating extracellular vesicle population. With this,

in mind, and extrapolating from previous data [27], we can anticipate that PDEVs should be able to deliver to tumors with dimensions of roughly 0.4 cm^3 (the total accumulation depends on the tumor mass) about half of the dose of the molecules loaded in the total population. This consideration becomes particularly relevant when considering potential future applications of PDEVs for drug delivery, such as chemotherapy agents. Assuming a drug encapsulation efficiency similar to that achieved with ICG, a reference value of approximately 5 µmol/Kg might be considered for the agent delivery at a dose of 3.3E09/Kg in mice. In this condition, we expect that we can deliver up to 150 nmol of a chemotherapeutic agent to a tumor of 1 gram (wet weight). Considering that doxorubicin when systemically administered reach a Cmax of 3 nmol/g within the tumor [62], with PDEVs it would be possible to deliver a dose of about 2 orders of magnitude higher than what can be achieved through systemic treatment of the same drug. The combination of high concentration with selective delivery to the cancerous tissue is expected to significantly increase the efficacy of well-established anti-neoplastic agents when loaded into PDEVs. The establishment of a freeze-drying protocol to preserve the size, dispersion and integrity of PDEVs pave the way to future broader clinical applications of PDEVs beyond intraoperative imaging. Indeed, lyophilization is an effective preservation method that offers long-term stabilization of EV excipients in favorable storage conditions in terms of clinical application and transportation, circumventing issues related to deep-freezing. The feasibility of preparing batches of PDEVs when patients are in good health condition opens the door for subsequent repeated administrations of drugs or diagnostics throughout the disease course. This advantageous aspect, combined with the good safety profile, easy production in hospital facilities, and simple storage, all contribute to the potential promotion of autologous EVs in delivering anti-neoplastic agents.

All in all, this manuscript reports a series of pharmacological data necessary for the use of autologous extracellular vesicles in humans for theranostics purposes, and as such, it presents certain limitations. For instance, we only focused on EVs isolated from patients with CRC. This choice stems from the design of the AUTOTERANOST protocol, which was developed in collaboration with the HPB Surgery and Liver Transplantation Department of the National Cancer Institute of Milan. The protocol stems from several years of preclinical studies on the biodistribution of tumor-derived EVs, and aims to apply the intraoperative imaging protocol clinically, starting with a trial phase in CRC patients. This justifies the use of EVs isolated from these patients, following informed consent and approval of the protocol by the INT ethics committee. However, despite the fact that CRC-derived vesicles are the

most well-characterized by our research group, we have previously investigated whether the homing capacity to tumors is a characteristic shared by EVs derived from other types of tumors as well [20, 25-28, 39, 41, 63]. Our research has demonstrated that not only is this capacity common to vesicles produced by all the tumors we have studied, but it is also a feature maintained across species [20], allowing EVs to home in on heterologous tumors generated in other species. The tumors considered include lung cancer [20], breast cancer [26], and central nervous system tumors, as recently shown using extracellular vesicles from canine glioblastoma patients [41]. Given that in all previously studied cases we did not observe differences in tumor accumulation times, we are confident that the pharmacokinetics determined in the current manuscript are also representative of EVs produced by other tumors.

For the same reasons, in this study we decided to use syngeneic murine models to evaluate the pharmacokinetics of PDEVs, instead of the humanized models like the Patient Derived Xenografts (PDX). Indeed, while in the initial study that led to the development of the AUTOTERANOST protocol, we investigated and verified the homing of PDEVs in PDX models generated using tumors resected from the same patients from whom we isolated the EVs, which were then implanted in immunodeficient mice [39], in following experiments, we verified that PDEVs could be used to deliver diagnostic molecules to murine tumors implanted in immunocompetent animals [27, 41]. Given that the affinity of PDEVs has proven consistent across various tumor models tested previously, including the PDX, we selected the syngeneic murine models, because the model presents fewer ethical issues compared to PDX models - which requires transplanting biopsy tissue from the patient to a mouse - and it involves the use of immunocompetent animals, providing a more physiologically relevant environment. While this might initially appear to be a limitation of the study, our previous studies did not show differences in the tumor-homing behavior of the PDEVs when administered to a PDX or to a syngeneic mouse, thus we are confident that the model we used closely mimics the pathophysiology likely to be encountered in patients during a clinical trial. This choice ensures that our results are more directly applicable to the clinical setting, where patients have fully functional immune systems.

Conclusions

The ability to selectively target diagnostic and therapeutic agents directly to neoplastic tissue has long been a key goal in cancer research. Despite various approaches, achieving this

goal has often fallen short of expectations. Thus, the need for biocompatible tools for the selective delivery of anti-tumor drugs or diagnostic agents remains a significant unmet need in both clinical and preclinical research. This paper builds on previous research demonstrating that tumor-derived EVs inherently possess tropism for tumors, a feature with significant clinical potential. Our study addressed the principal challenges that must be overcome to accelerate the clinical use of autologous PDEVs. We developed the ONCOGREEN manufacturing protocol, which identifies the optimal posology for effective tumor identification through PDEVs-ICG accumulation. We verified the safety of the approach and, to tackle the challenge of producing a sufficient quantity of PDEVs for clinical use, we have implemented a plasmapheresis procedure. This technique ensures the generation of an adequate number of EVs, which is essential for achieving therapeutic efficacy. The plasmapheresis process is compatible with hospital admission and cancer surgery protocols, allowing for seamless integration into patient care workflows without significant disruptions or the need for extensive modifications. Additionally, the procedure aligns well with the existing infrastructure and equipment found in the pharmacy of medium-sized hospitals. This compatibility means that hospitals can adopt this technology without the need for specialized equipment or extensive training, facilitating widespread implementation. Looking forward, establishing in-house PDEVs production could transform cancer treatment management. By creating an internal production service, hospitals can ensure a consistent and reliable supply of PDEVs tailored to their patients' needs. This self-sufficiency reduces dependence on external suppliers and enhances the precision and personalization of cancer therapy. Beyond surgery, the theranostic potential of PDEVs is promising. Should PDEVs demonstrate sufficient specificity and sensitivity in vivo, their use could be expanded to include contrast agents (e.g., gadolinium or iohexol) or targeted drugs. This selective affinity would enhance therapeutic efficacy, minimize systemic side effects, and potentially reduce inflammation outside the tumor area. By democratizing access to advanced therapeutic options, we can significantly improve cancer care, offering more effective and targeted treatments, and paving the way for widespread adoption of innovative oncological therapies.

Abbreviations

ALP: alkaline phosphatase; ALT: alanine aminotransferase; CFSE: carboxyfluorescein succinimidyl ester; CK: creatine kinase; CRC: colorectal cancer; Cryo-EM: cryo electron microscopy; EVs: extracellular vesicles; GLDH: glutamate dehydrogenase; ICG:

indocyanine green; i.v.: intravenous; LDH: lactate dehydrogenase; MED: minimum effective dose; NIR: near-infrared; NTA: nanoparticle tracking analysis; PDEVs: patient-derived extracellular vesicles; PDX: patient derived xenografts; PT: prothrombin time; aPTT: partial thromboplastin time; TSG101: Tumor Susceptibility Gene – 101.

Funding

The research leading to the results has received funding from AIRC under IG 2020 - ID.24914 project to P.C., and European Union — NextGenerationEU (PNRR M4C2-Investimento 1.4-CN00000041-23 PNRR_CN3RNA_SPOKE8 to P.C. and F.C. and PNRR_CN3RNA_SPOKE9 to P.C.).

Institutional Review Board Statement

All animal experimentation was carried out in accordance with the Animal Research: Reporting of in Vivo Experiments (ARRIVE) guidelines and the European Guidelines for Animal Care. All animal experiments were approved by the Italian Ministry of Research and University, permission number: 214/2020. Blood draws and plasmapheresis procedures on CRC patients were approved by the Ethics Committee of the National Cancer Institute of Milan (Aut. INT 244/20).

CRediT authorship contribution statement

Conceptualization, A.V., D.C. and P.C.; Formal analysis, Z.D., E.C. A.V. and P.C.; Funding acquisition, V.M., P.C. ans F.C.; Investigation, D.C., A.V., Z.D., E.C.; Methodology, D.C., A.V., Z.D., E.C., S.R., S.V., F.Sh., L.C., A.O., A.G., M.R, N.S., M.M., C.R., F.S., S.P., M.G., V.L., N.S., F.A. and P.C.; Writing—original draft, A.V. and P.C.; Writing—review & editing, A.V., P.C. and E.B. All authors have read and agreed to the published version of the manuscript.

Declaration of Competing Interest

The authors declare that they have no known competing financial interests or personal relationships that could have appeared to influence the work reported in this paper.

Data Availability

Data will be made available on request.

References

1. Liu X, Cheng Y, Mu Y, Zhang Z, Tian D, Liu Y, et al. Diverse drug delivery systems for the enhancement of cancer immunotherapy: an overview. Front Immunol. 2024; 15: 1328145.
2. Ding L, Agrawal P, Singh SK, Chhonker YS, Sun J, Murry DJ. Polymer-Based Drug Delivery Systems for Cancer Therapeutics. Polymers (Basel). 2024; 16.
3. Park D, Lee SJ, Park JW. Aptamer-Based Smart Targeting and Spatial Trigger-Response Drug-Delivery Systems for Anticancer Therapy. Biomedicines. 2024; 12.
4. Yan S, Na J, Liu X, Wu P. Different Targeting Ligands-Mediated Drug Delivery Systems for Tumor Therapy. Pharmaceutics. 2024; 16.
5. Li R, He Y, Zhang S, Qin J, Wang J. Cell membrane-based nanoparticles: a new biomimetic platform for tumor diagnosis and treatment. Acta Pharmaceutica Sinica B. 2018; 8: 14-22.
6. Khosravi N, Pishavar E, Baradaran B, Oroojalian F, Mokhtarzadeh A. Stem cell membrane, stem cell-derived exosomes and hybrid stem cell camouflaged nanoparticles: A promising biomimetic nanoplatforms for cancer theranostics. J Control Release. 2022; 348: 706-22.
7. Zhai Y, Su J, Ran W, Zhang P, Yin Q, Zhang Z, et al. Preparation and Application of Cell Membrane-Camouflaged Nanoparticles for Cancer Therapy. Theranostics. 2017; 7: 2575-92.
8. Pan H, Yang S, Gao L, Zhou J, Cheng W, Chen G, et al. At the crossroad of nanotechnology and cancer cell membrane coating: Expanding horizons with engineered nanoplatforms for advanced cancer therapy harnessing homologous tumor targeting. Coord Chem Rev. 2024; 506: 215712.
9. Shao M, Lopes D, Lopes J, Yousefiasl S, Macário-Soares A, Peixoto D, et al. Exosome membrane-coated nanosystems: Exploring biomedical applications in cancer diagnosis and therapy. Matter. 2023; 6: 761-99.
10. Li X, Lin Y, Yang Z, Guan L, Wang Z, Liu A, et al. Cancer cell membrane biomimetic nanosystem for homologous targeted dual-mode imaging and combined therapy. J Colloid Interface Sci. 2023; 652: 770-9.
11. Wortzel I, Dror S, Kenific CM, Lyden D. Exosome-Mediated Metastasis: Communication from a Distance. Dev Cell. 2019; 49: 347-60.
12. Wang Z, Wang Q, Qin F, Chen J. Exosomes: a promising avenue for cancer diagnosis beyond treatment. Front Cell Dev Biol. 2024; 12: 1344705.
13. Buzas EI. The roles of extracellular vesicles in the immune system. Nature Reviews Immunology. 2023; 23: 236-50.
14. Cano A, Ettcheto M, Bernuz M, Puerta R, de Antonio EE, Sánchez-López E, et al. Extracellular vesicles, the emerging mirrors of brain physiopathology. Int J Biol Sci. 2023; 19: 721.
15. Elsharkasy OM, Nordin JZ, Hagey DW, de Jong OG, Schiffelers RM, Andaloussi SEL, et al. Extracellular vesicles as drug delivery systems: Why and how? Adv Drug Del Rev. 2020; 159: 332-43.
16. van der Meel R, Sulheim E, Shi Y, Kiessling F, Mulder WJM, Lammers T. Smart cancer nanomedicine. Nature nanotechnology. 2019; 14: 1007-17.
17. Wiklander OPB, Nordin JZ, O'Loughlin A, Gustafsson Y, Corso G, Mäger I, et al. Extracellular vesicle in vivo biodistribution is determined by cell source, route of administration and targeting. Journal of extracellular vesicles. 2015; 4: 26316-.
18. Edelmann MJ, Kima PE. Current understanding of extracellular vesicle homing/tropism. Zoonoses (Burlington, Mass). 2022; 2: 14.
19. Tian Y, Li S, Song J, Ji T, Zhu M, Anderson GJ, et al. A doxorubicin delivery platform using engineered natural membrane vesicle exosomes for targeted tumor therapy. Biomaterials. 2014; 35: 2383-90.
20. Garofalo M, Villa A, Crescenti D, Marzagalli M, Kuryk L, Limonta P, et al. Heterologous and cross-species tropism of cancer-derived extracellular vesicles. Theranostics. 2019; 9: 5681-93.
21. Hoshino A, Costa-Silva B, Shen T-L, Rodrigues G, Hashimoto A, Tesic Mark M, et al. Tumour exosome integrins determine organotropic metastasis. Nature. 2015; 527: 329-35.
22. Rodrigues G, Hoshino A, Kenific CM, Matei IR, Steiner L, Freitas D, et al. Tumour exosomal CEMIP protein promotes cancer cell colonization in brain metastasis. Nat Cell Biol. 2019; 21: 1403-12.
23. Bie N, Yong T, Wei Z, Gan L, Yang X. Extracellular vesicles for improved tumor accumulation and penetration. Adv Drug Deliv Rev. 2022; 188: 114450.
24. Pavon LF, Sibov TT, de Souza AV, da Cruz EF, Malheiros SMF, Cabral FR, et al. Tropism of mesenchymal stem cell toward CD133(+) stem cell of glioblastoma in vitro and promote tumor proliferation in vivo. Stem Cell Res Ther. 2018; 9: 310-.

25. Garofalo M, Villa A, Rizzi N, Kuryk L, Rinner B, Cerullo V, et al. Extracellular vesicles enhance the targeted delivery of immunogenic oncolytic adenovirus and paclitaxel in immunocompetent mice. Journal of Controlled Release. 2019; 294: 165-75.
26. Garofalo M, Villa A, Brunialti E, Crescenti D, Dell'Omo G, Kuryk L, et al. Cancer-derived EVs show tropism for tissues at early stage of neoplastic transformation. Nanotheranostics. 2021; 5: 1-7.
27. Vincenti S, Villa A, Crescenti D, Crippa E, Brunialti E, Shojaei-Ghahrizjani F, et al. Increased Sensitivity of Computed Tomography Scan for Neoplastic Tissues Using the Extracellular Vesicle Formulation of the Contrast Agent Iohexol. Pharmaceutics. 2022; 14: 2766.
28. Garofalo M, Saari H, Somersalo P, Crescenti D, Kuryk L, Aksela L, et al. Antitumor effect of oncolytic virus and paclitaxel encapsulated in extracellular vesicles for lung cancer treatment. Journal of Controlled Release. 2018; 283: 223-34.
29. Liu S, Wu X, Chandra S, Lyon C, Ning B, Jiang L, et al. Extracellular vesicles: Emerging tools as therapeutic agent carriers. Acta pharmaceutica Sinica B. 2022; 12: 3822-42.
30. Zhang P, Zhang L, Qin Z, Hua S, Guo Z, Chu C, et al. Genetically Engineered Liposome-like Nanovesicles as Active Targeted Transport Platform. Advanced Materials. 2017; 30.
31. He G, Liu J, Yu Y, Wei S, Peng X, Yang L, et al. Revisiting the advances and challenges in the clinical applications of extracellular vesicles in cancer. Cancer Lett. 2024; 593: 216960.
32. Kreger BT, Dougherty AL, Greene KS, Cerione RA, Antonyak MA. Microvesicle Cargo and Function Changes upon Induction of Cellular Transformation. The Journal of biological chemistry. 2016; 291: 19774-85.
33. Kreger BT, Johansen ER, Cerione RA, Antonyak MA. The Enrichment of Survivin in Exosomes from Breast Cancer Cells Treated with Paclitaxel Promotes Cell Survival and Chemoresistance. Cancers (Basel). 2016; 8: 111.
34. Huang Y, Kanada M, Ye J, Deng Y, He Q, Lei Z, et al. Exosome-mediated remodeling of the tumor microenvironment: From local to distant intercellular communication. Cancer Lett. 2022; 543: 215796.
35. Kalluri R, McAndrews KM. The role of extracellular vesicles in cancer. Cell. 2023; 186: 1610-26.
36. Sheta M, Taha EA, Lu Y, Eguchi T. Extracellular Vesicles: New Classification and Tumor Immunosuppression. Biology (Basel). 2023; 12.
37. Zhou X, Jia Y, Mao C, Liu S. Small extracellular vesicles: Non-negligible vesicles in tumor progression, diagnosis, and therapy. Cancer Lett. 2024; 580: 216481.
38. Ming-Kun C, Zi-Xian C, Mao-Ping C, Hong C, Zhuang-Fei C, Shan-Chao Z. Engineered extracellular vesicles: A new approach for targeted therapy of tumors and overcoming drug resistance. Cancer Commun (Lond). 2024; 44: 205-25.
39. Villa A, Garofalo M, Crescenti D, Rizzi N, Brunialti E, Vingiani A, et al. Transplantation of autologous extracellular vesicles for cancer-specific targeting. Theranostics. 2021; 11: 2034-47.
40. Ciana P, Garofalo M, Villa AM, Mazzaferro V, Maggi A. Pat. WO2020240494A1: Extracellular vesicles for delivering therapeutic or diagnostic drugs. 2022.
41. Villa A, De Mitri Z, Vincenti S, Crippa E, Castiglioni L, Gelosa P, et al. Canine glioblastoma-derived extracellular vesicles as precise carriers for glioblastoma imaging: Targeting across the blood-brain barrier. Biomed Pharmacother. 2024; 172: 116201.
42. Welsh JA, Goberdhan DCI, O'Driscoll L, Buzas EI, Blenkiron C, Bussolati B, et al. Minimal information for studies of extracellular vesicles (MISEV2023): From basic to advanced approaches. J Extracell Vesicles. 2024; 13: e12404.
43. Garofalo M, Villa A, Rizzi N, Kuryk L, Rinner B, Cerullo V, et al. Extracellular vesicles enhance the targeted delivery of immunogenic oncolytic adenovirus and paclitaxel in immunocompetent mice. J Control Release. 2019; 294: 165-75.
44. Garofalo M, Saari H, Somersalo P, Crescenti D, Kuryk L, Aksela L, et al. Antitumor effect of oncolytic virus and paclitaxel encapsulated in extracellular vesicles for lung cancer treatment. J Control Release. 2018; 283: 223-34.
45. Skotland T, Iversen TG, Llorente A, Sandvig K. Biodistribution, pharmacokinetics and excretion studies of intravenously injected nanoparticles and extracellular vesicles: Possibilities and challenges. Adv Drug Del Rev. 2022; 186: 114326.

46. Alander JT, Kaartinen I, Laakso A, Pätilä T, Spillmann T, Tuchin VV, et al. A review of indocyanine green fluorescent imaging in surgery. Int J Biomed Imaging. 2012; 2012: 940585-.
47. Boni L, David G, Mangano A, Dionigi G, Rausei S, Spampatti S, et al. Clinical applications of indocyanine green (ICG) enhanced fluorescence in laparoscopic surgery. Surg Endosc. 2015; 29: 2046-55.
48. Parada N, Romero-Trujillo A, Georges N, Alcayaga-Miranda F. Camouflage strategies for therapeutic exosomes evasion from phagocytosis. Journal of advanced research. 2021; 31: 61-74.
49. Nguyen VD, Kim HY, Choi YH, Park J-O, Choi E. Tumor-derived extracellular vesicles for the active targeting and effective treatment of colorectal tumors in vivo. Drug Deliv. 2022; 29: 2621-31.
50. Escudier B, Dorval T, Chaput N, André F, Caby M-P, Novault S, et al. Vaccination of metastatic melanoma patients with autologous dendritic cell (DC) derived-exosomes: results of thefirst phase I clinical trial. J Transl Med. 2005; 3: 10-.
51. Morse MA, Garst J, Osada T, Khan S, Hobeika A, Clay TM, et al. A phase I study of dexosome immunotherapy in patients with advanced non-small cell lung cancer. J Transl Med. 2005; 3: 9-.
52. Sercombe L, Veerati T, Moheimani F, Wu SY, Sood AK, Hua S. Advances and challenges of liposome assisted drug delivery. Front Pharmacol. 2015; 6: 286.
53. Drummond DC, Noble CO, Guo Z, Hong K, Park JW, Kirpotin DB. Development of a highly active nanoliposomal irinotecan using a novel intraliposomal stabilization strategy. Cancer Res. 2006; 66: 3271-7.
54. Sugiyama T, Asai T, Nedachi YM, Katanasaka Y, Shimizu K, Maeda N, et al. Enhanced active targeting via cooperative binding of ligands on liposomes to target receptors. PLoS One. 2013; 8: e67550.
55. Soprano E, Polo E, Pelaz B, Del Pino P. Biomimetic cell-derived nanocarriers in cancer research. J Nanobiotechnology. 2022; 20: 538.
56. Herrmann IK, Wood MJA, Fuhrmann G. Extracellular vesicles as a next-generation drug delivery platform. Nat Nanotechnol. 2021; 16: 748-59.
57. Schubert A, Boutros M. Extracellular vesicles and oncogenic signaling. Mol Oncol. 2021; 15: 3-26.
58. Besse B, Charrier M, Lapierre V, Dansin E, Lantz O, Planchard D, et al. Dendritic cell-derived exosomes as maintenance immunotherapy after first line chemotherapy in NSCLC. Oncoimmunology. 2015; 5: e1071008-e.
59. Lightner AL, Sengupta V, Qian S, Ransom JT, Suzuki S, Park DJ, et al. Bone Marrow Mesenchymal Stem Cell-Derived Extracellular Vesicle Infusion for the Treatment of Respiratory Failure From COVID-19: A Randomized, Placebo-Controlled Dosing Clinical Trial. Chest. 2023; 164: 1444-53.
60. Matsumoto A, Takahashi Y, Ogata K, Kitamura S, Nakagawa N, Yamamoto A, et al. Phosphatidylserine-deficient small extracellular vesicle is a major somatic cell-derived sEV subpopulation in blood. Iscience. 2021; 24.
61. Shimizu A, Sawada K, Kobayashi M, Yamamoto M, Yagi T, Kinose Y, et al. Exosomal CD47 plays an essential role in immune evasion in ovarian cancer. Mol Cancer Res. 2021; 19: 1583-95.
62. Stallard S, Morrison JG, George WD, Kaye SB. Distribution of doxorubicin to normal breast and tumour tissue in patients undergoing mastectomy. Cancer Chemother Pharmacol. 1990; 25: 286-90.
63. Garofalo M, Villa A, Rizzi N, Kuryk L, Mazzaferro V, Ciana P. Systemic Administration and Targeted Delivery of Immunogenic Oncolytic Adenovirus Encapsulated in Extracellular Vesicles for Cancer Therapies. Viruses. 2018; 10: 558.

Tables

Table 1. Number and dimension distribution of PDEVs isolated from blood draws of two CRC patients. SD: Standard deviation. D10: diameter (nm) of the particles that is the 10th percentile. D50: diameter (nm) of the particles that is the 50th percentile. D90: diameter (nm) of the particles that is the 90th percentile

Value	PATIENT 1	PATIENT 2
Mean	196.1	174.2
Mode	123.3	132.3
SD	83.8	80.1
D10	119.8	111.7
D50	171.6	149.0
D90	309.2	265.2
EV/ml	4.2 E10	3.1 E10

Table 2. Plasma coagulation times and bone marrow analysis, made on samples of C57BL/6 mice collected 1 hour after treatments with Vehicle, or two different doses of syngeneic EVs (3.3E09 EVs/Kg and 3.3E10 EVs/Kg). *N*=8; *: pVal<0.05 vs Vehicle by t -test.

Parameters	Vehicle	3.3E09 EVs/kg	3.3E10 EVs/kg
**fibrinogen (mg/dL)**	111.00 ± 18.94	92.50 ± 25.29 *	58.75± 5.77 *
**PT (sec)**	71.65 ± 59.45	131.57 ± 68.37 *	136.27 ± 62.30 *
**APTT (sec)**	96.40 ± 51.37	197.55 ± 52.45 *	250.00 ± 0 *
**Myeloid-erythroid ratio**	1.45 ± 0.14	1.52 ± 0.13	0.17± 0.11 *
**Proliferative erythoid pool (%)**	12.11 ±1.14	16.56± 1.29	14.76 ± 2.13
**Proliferative myeloid pool (%)**	12.19±1.50	18.98± 0.77	23.02± 1.14 *
**Lymphocytes%**	9.78± 1.23	10.14± 0.60	8.31± 0.83

Table 3. Clinical chemistry results. ALT: Alanine Transaminase; ALP: Alkaline Phosphatase; Chol: cholesterol; CK: Creatine Kinase; LDH: Lactate Dehydrogenase; GLDH: Glutamate dehydrogenase. Reference values for C57BL/6 mice are reported in Supplementary Table 2.

	Clinical chemistry – 24h			Clinical chemistry – 10 days		
**Parameters**	**Vehicle**	**3.3E09 EVs/Kg**	**3.3E10 EVs/Kg**	**Vehicle**	**3.3E09 EVs/Kg**	**3.3E10 EVs/Kg**
**Glucose (mg/dL)**	370.00 ± 84.90	348.50 ± 71.11	333.00 ± 52.42	421.00 ± 87.07	267.50 ± 36.76	354.00 ± 76.22
**Urea (mg/dL)**	71.88 ± 5.89	76.95 ± 3.07	72.28 ± 5.77	79.15 ± 4.14	77.64 ± 4.90	77.76 ± 3.62
**Creatinine (mg/dl)**	0.30 ± 0.04	0.37 ± 0.05	0.40 ± 0.04	0.39 ± 0.03	0.34± 0.05	0.38 ± 0.02
**Tot. proteins (g/dL)**	6.56 ± 0.28	6.73 ± 0.15	6.52 ± 0.25	6.88 ± 0.47	6.48 ± 0.22	6.80 ± 0.26
**Albumin (g/dL)**	3.76 ± 0.19	4.03 ± 0.13	3.81± 0.19	4.23 ± 0.28	3.89 ± 0.12	3.89 ± 0.12
**ALT (U/L)**	51.50 ± 17.44	180.60 ± 82.83	267.25 ± 58.96	324.00 ± 140.99	102.16± 38.50	263.00 ± 79.18
**GLDH (U/L)**	54.50 ± 20.99	86.99 ± 19.54	89.93 ± 36.00	119.00± 19.54	48.00± 18.02	64.00± 18.13
**ALP (U/L)**	113.78 ± 29.15	134.64 ± 25.09	130.68 ± 18.75	119.21 ± 26.19	170.75 ± 23.83	146.03 ± 30.55
**Chol (mg/dL)**	119.17 ± 17.83	115.56 ± 7.06	110.08 ± 5.17	114.72 ± 13.04	107.08 ± 7.34	114.33 ± 3.66
**CK (U/L)**	10979 ± 8809	6953.00 ± 3146.96	4523.00 ± 739.90	7064.12 ± 3263.32	4946.66 ± 2691.00	8294.33 ± 4718.88
**LDH (U/L)**	2536.00 ± 864.88	2013.00 ± 584.15	2060.00 ± 388.56	2227.66 ± 616.71	1561.66 ± 421.83	1225.00 ± 555.88

Table 4. Hematology on peripheral blood. RBC: red blood cells; WBC: white blood cells. *: pVal<0.01 vs Vehicle; #: pVal<0.01 vs 10^9 EVs. Reference values for C57BL/6 mice are reported in Supplementary Table 2.

	Hematology on peripheral blood – 24h			Hematology on peripheral blood – 10 days		
**Parameters**	**Vehicle**	**3.3E09 EVs/Kg**	**3.3E10 EVs/Kg**	**Vehicle**	**3.3E09 EVs/Kg**	**3.3E10 EVs/Kg**
**Hemoglobin (g/dL)**	10.66 ±1.48	10.91 ±1.01	12.09 ± 0.45	10.14 ± 1.11	11.90± 0.21	10.60± 1.32
**Mean corpuscular hemoglobin (pg)**	15.20 ± 0.35	14.78 ±0.32	14.65 ± 0.32	14.77 ± 0.32	14.70± 0.17	15.56 ± 0.39
**Mean corpuscular hemoglobin concentration (g/dL)**	34.34 ± 53.07	32.91± 1.03	32.75 ± 0.47	33.88 ± 0.84	33.01 ± 0.48	33.95 ± 0.99
**RBC(10^6/µl)**	7.17 ± 1.01	7.46 ± 0.77	8.25 ± 0.31	6.65 ± 0.77	8.08 ± 0.13	6.96 ± 0.90
**WBC (10^3/µl)**	5.36 ± 1.03	4.19 ± 0.56	5.23 ± 0.91	8.06 ± 2.91	5.85 ± 0.73*	4.08 ± 1.07*
**Neutrophils%**	9.34 ± 1.64	10.01± 2.20	9.24 ±1.07	6.42 ± 1.78	14.96 ± 6.03#	4.97 ± 0.55
**Eosinophils%**	1.21 ± 0.19	1.43 ± 0.32	1.16± 0.22	1.09 ± 0.14	1.43± 0.22	1.02 ± 0.24
**Basophils%**	0.55 ± 0.20	0.33 ± 0.14	0.24± 0.09	0.20 ± 0.08	0.23± 0.08	0.34 ± 0.13
**Lymphocytes%**	82.33 ± 1.93	77.86 ± 1.88	78.54± 2.38	79.93 ± 4.65	73.53 ± 6.37	79.89 ± 3.31
**Monocytes%**	9.56 ± 1.53	10.38 ± 1.51	10.81± 2.18	12.35 ± 3.52	9.85 ± 2.07	8.79 ± 3.59
**Platelet (10^3/µl)**	211 ± 126	213 ± 57	210 ± 58	216.25 ± 49.86	412.14 ± 248.79	273.88 ± 72.28

Table 5. Dimension profiles of the PDEVs obtained from four CRC patients using the plasmapheresis protocol. SD: Standard deviation. D_{10}: diameter (nm) of the particles that is the 10th percentile. D_{50}: diameter (nm) of the particles that is the 50th percentile. D_{90}: diameter (nm) of the particles that is the 90th percentile.

Value	PATIENT 1	PATIENT 2	PATIENT 3	PATIENT 4
Mean	183,2	192,0	196,2	190,5
Mode	119,5	130,2	122,7	123,6
SD	80,4	90,4	98,5	72,0
D10	101,3	115,1	100,7	121,6
D50	160,0	167,4	198,2	161,5
D90	279,6	315,5	323,2	254,6
EV/ml	2,45 E10	8,4 E10	7,58 E10	5,36 E10

Figures

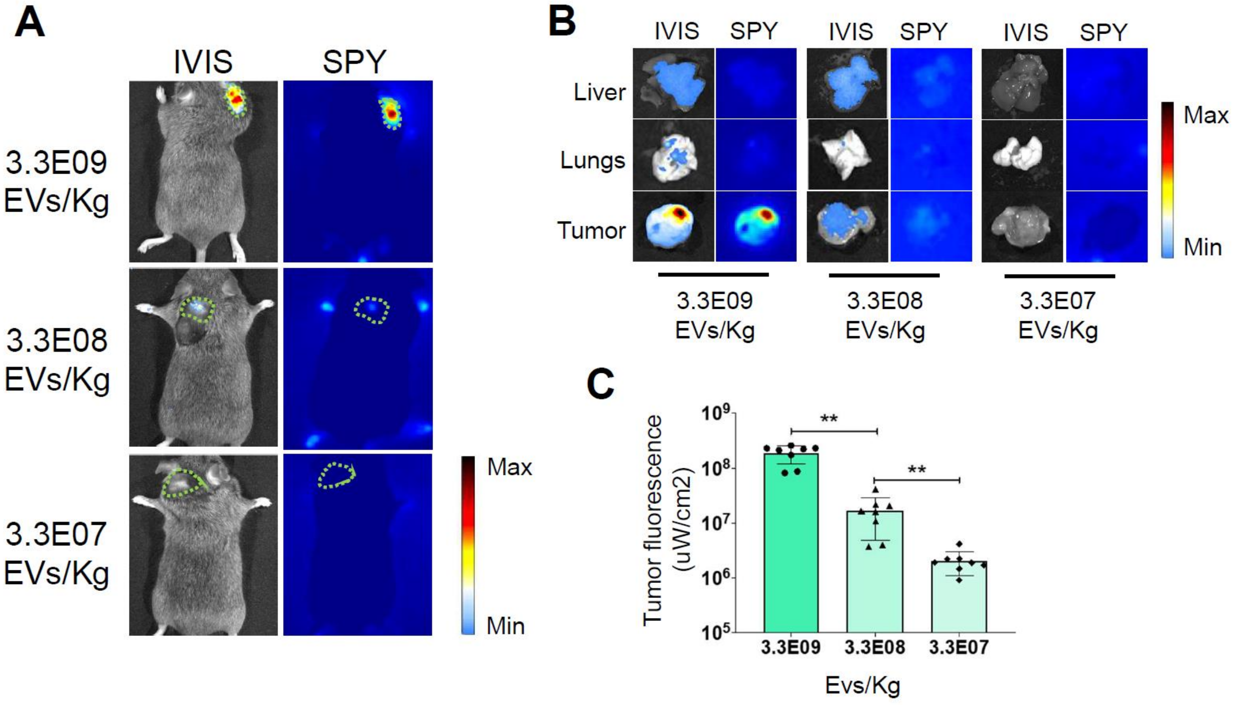

Figure 1. Determination of the Minimum Effective Dose. Representative images of ICG fluorescence *in vivo* (A) and *ex vivo* in the tumor, lung, and liver (B), obtained with the IVIS Spectrum Imaging System and the SPY Elite intraoperative imaging device. The tumor margins in the *in vivo* pictures are highlighted by the green dotted line. In the color scale, blue represents the minimum fluorescence signal, whereas red represents the maximum. Additional ex vivo images from other replicates are presented in Supplementary Figure 3. Mice bearing tumors were administered three dosages of nanoparticles – 3.3E09 EVs/Kg, 3.3E08 EVs/Kg, or 3.3E07 EVs/Kg - of the ONCOGREEN formulation. C) Quantification of the fluorescent signals in the tumors 24 hours after injection is presented in the graph; bars in the graph represent the average +/- S.E.M values of eight animals, *** $p < 0.001$, ** $p < 0.01$ calculated by one-way ANOVA followed by Bonferroni's test.

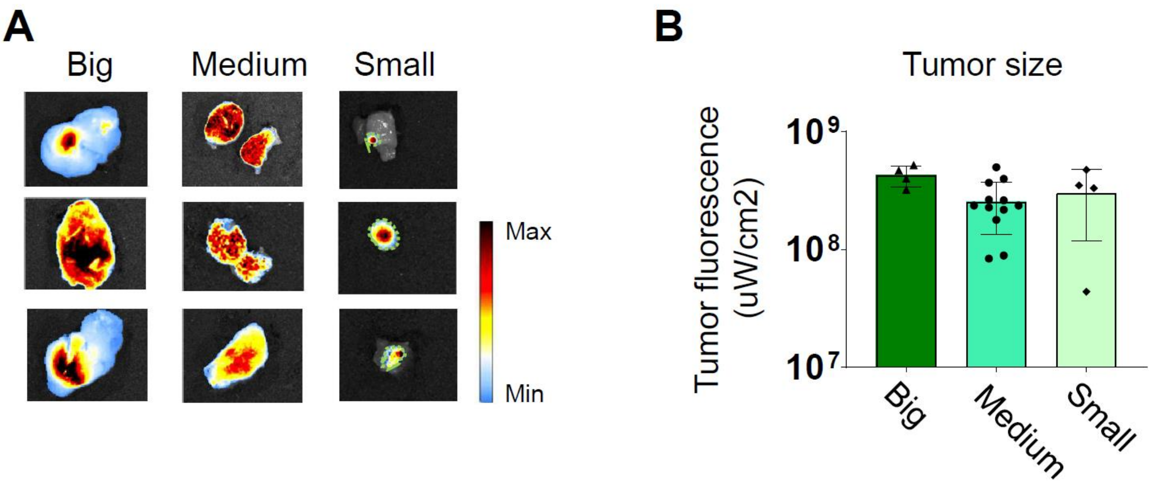

Figure 2. Independence of the average fluorescent signal intensity from for the tumor size. Three groups of mice bearing tumors of different sizes (small 0.1-0.25 cm³, medium 0.3-0.5 cm³, and large 0.7-1.0 cm³) were intravenously treated with 3.3E09 EVs/Kg of the ONCOGREEN formulation (MED). Mice were sacrificed 24 hours after treatment. A) Representative *ex vivo* images of ICG fluorescence. In big and medium tumors, fluorescence is emitted from the entire surface of the tumor, as indicated by the blue coloration representing the presence of a near-infrared fluorescent signal. The samples reported in the 'Small'-labelled column include the tumor collected together with surrounding healthy tissue: the tumor margins are highlighted by the green dotted line. Each individual picture in the panel represents a different mouse. In the color scale, blue represents the minimum fluorescence signal, whereas red represents the maximum. B) Quantification of the tumor fluorescent signal divided by the total tumor area.

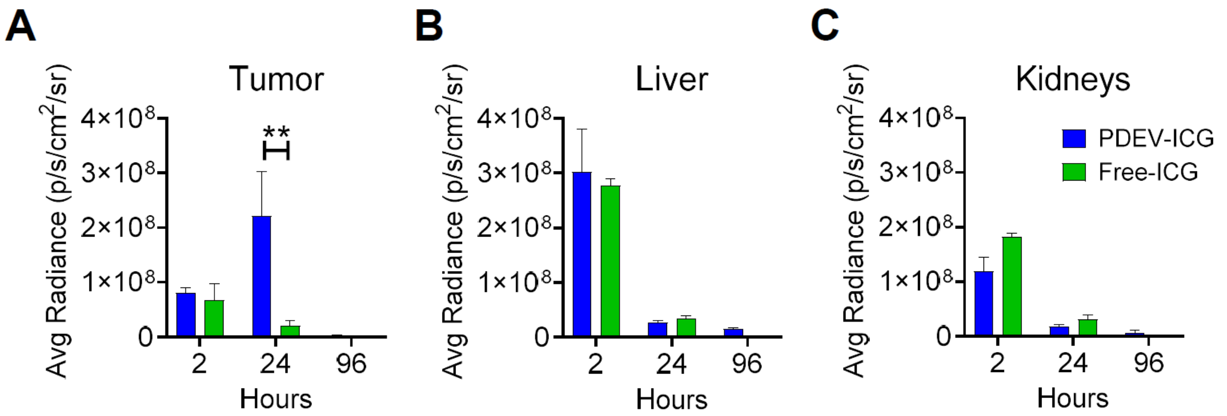

Figure 3. Kinetics of the biodistribution of EV-formulated ICG in tumor-bearing mice. Three groups of 4 mice each carrying syngeneic 0.4 cm^3 MC38 tumors were administered a single MED of ONCOGREEN (3.3E09 EVs/Kg) or an equal dose of the standard formulation of free ICG (4 mg free ICG/Kg). The fluorescence biodistribution was analyzed ex vivo at 2, 24, and 96 hours using IVIS Spectrum Imaging. Other organs are depicted in Supplementary Figure 5. **:pVal < 0.01 with ANOVA test.

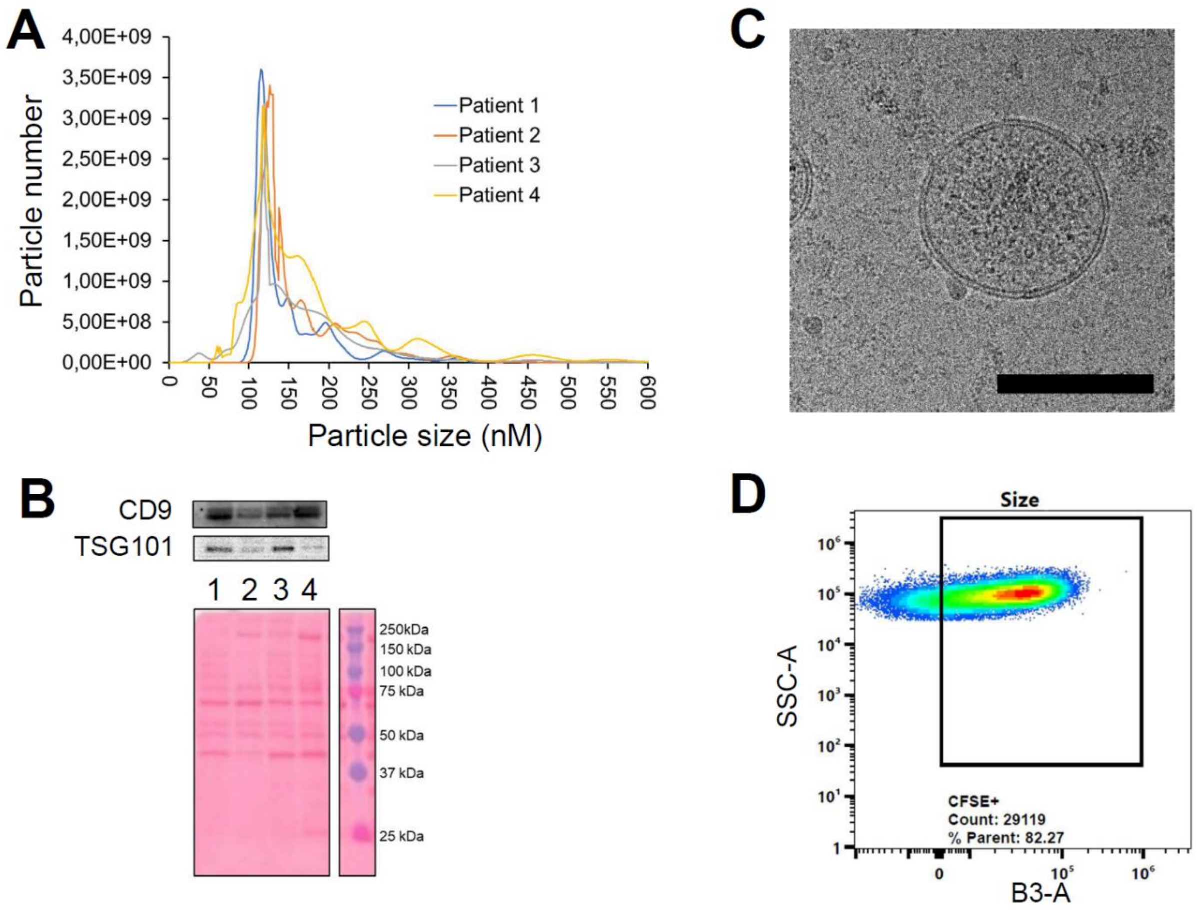

Figure 4. Characterization of EVs from colorectal cancer patients using the plasmapheresis protocol. The PDEVs exhibited size distribution, shape, EV-specific marker expression comparable to the PDEVs used in previous experiments. A) NTA of particle size distribution of PDEVs. The lines represent the mean of 5 readings. Details on the size distribution and concentration are provided in Table 4. B) Immunoblot analysis of TSG101 (47 KDa) expression in plasma-derived EVs from patients (lane 1: patient 1; lane 2: patient 2; lane 3: patient 3; lane 4: patient 4). C) Representative EV morphology and size obtained by cryo electron microscopy. Scale bar: 100 nm. D) Flow cytometry analysis of CFSE-labeled PDEVs showing the percentage (>80%) of CFSE-positive (CFSE+) vesicles. The cytogram depicts the side scatter (SSC)-A vs B3-A (green fluorescence triggering) used to trace the CFSE+ gate. CFSE, carboxyfluorescein succinimidyl ester; PDEVs, patient-derived extracellular vesicles.

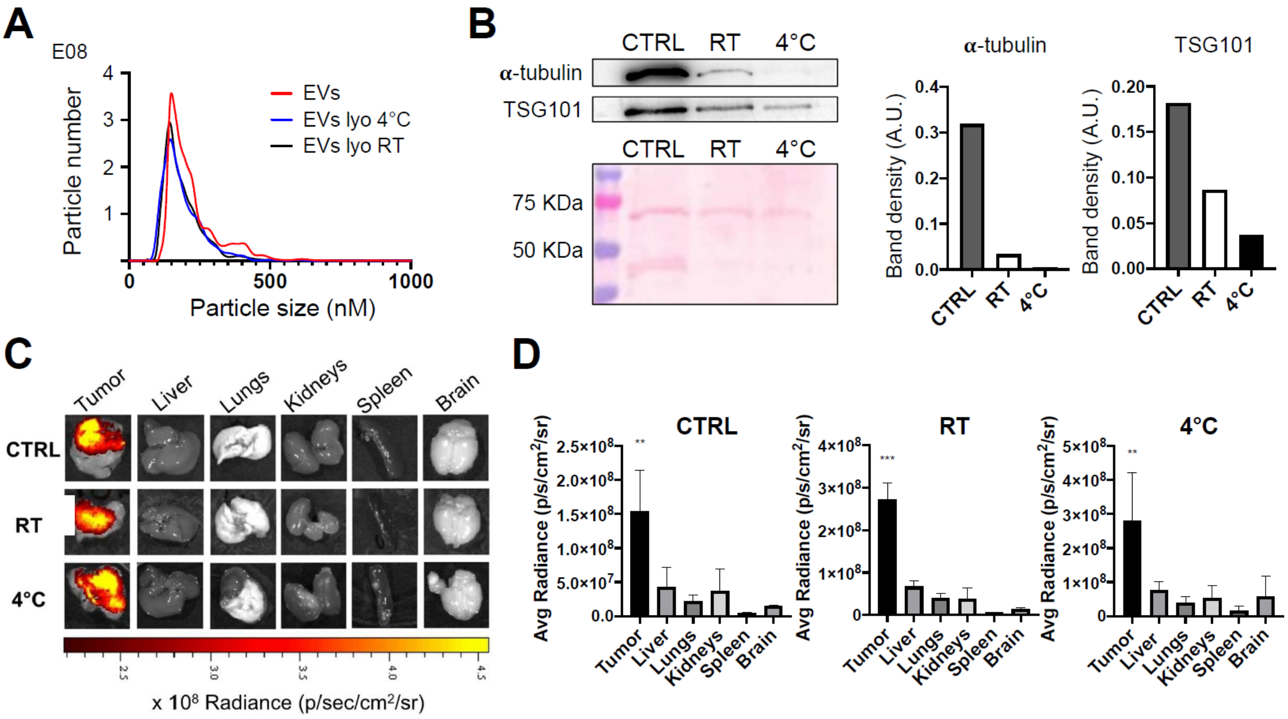

Figure 5. Characterization of lyophilized EVs. EVs derived from the MCF7 tumor cell line were freeze-dried and were subsequently stored at room temperature (RT) or 4°C for a duration of 4 months. A) Comparison of nanoparticle tracking analysis of MCF7-EVs and lyophilized MCF7-EVs kept at 4 °C and RT. The lyophilized vesicles showed similarities in terms of shape and size distribution when compared to non-lyophilized EVs (CTRL). B) Immunoblot analysis of EV marker proteins alpha-tubulin (50 KDa) and TSG101 (47 KDa) in both lyophilized and non-lyophilized MCF7 EVs. The graphs show the band density of proteins normalized for ponceau staining. C) Representative pseudocolored images display ex vivo ICG fluorescence in MC38 tumor-bearing mice. These images were captured 24h after the intravenous injection of EVs loaded with ICG, comparing lyophilized or non-lyophilized EVs. The color scale represents the fluorescence signal, with black indicating the minimum intensity and yellow indicating the maximum. D) The graphs show the ex vivo ICG fluorescence, 24h after treatment with lyophilized or non-lyophilized EVs. Additional In vivo imaging pictures for the RT group are reported in Supplementary Figure 12.

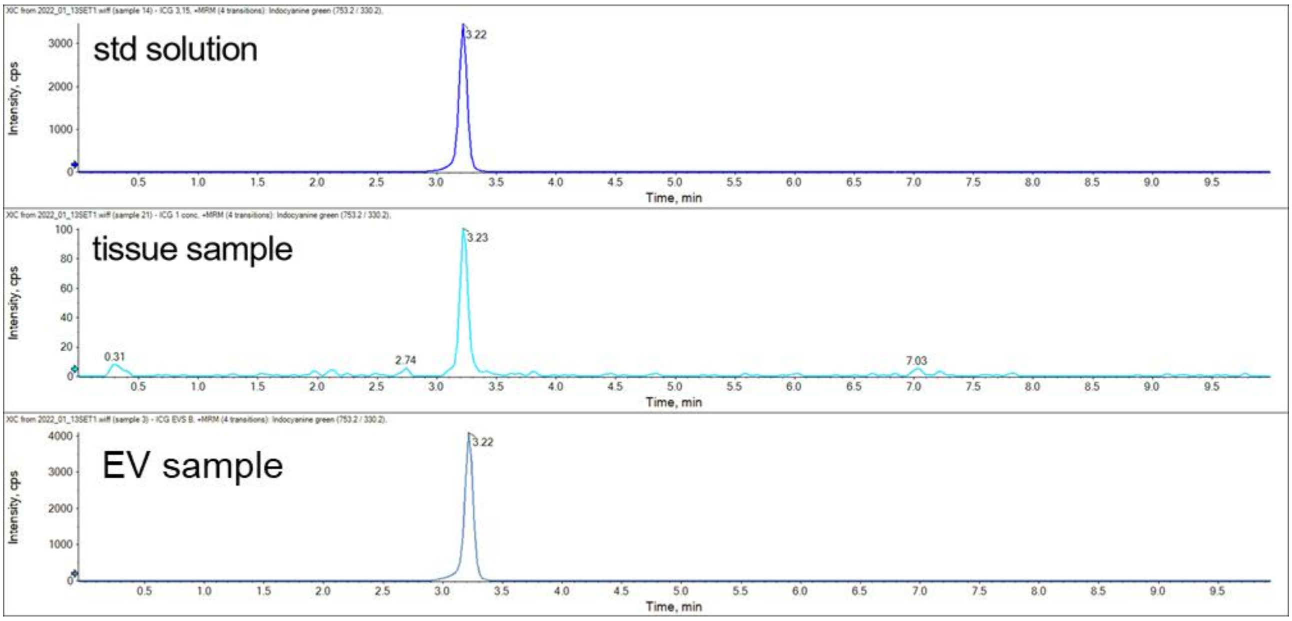

Supplementary Figure 1. High-Performance Liquid Chromatography-Mass Spectrometry (HPLC/MS) chromatogram showing the separation and detection of ICG in standard (std solution) and complex biological samples (tissue sample and EV sample). Peaks represent individual compounds separated based on their retention time, that have been analyzed by mass spectrometry. The intensity of each peak corresponds to the abundance of the respective analyte.

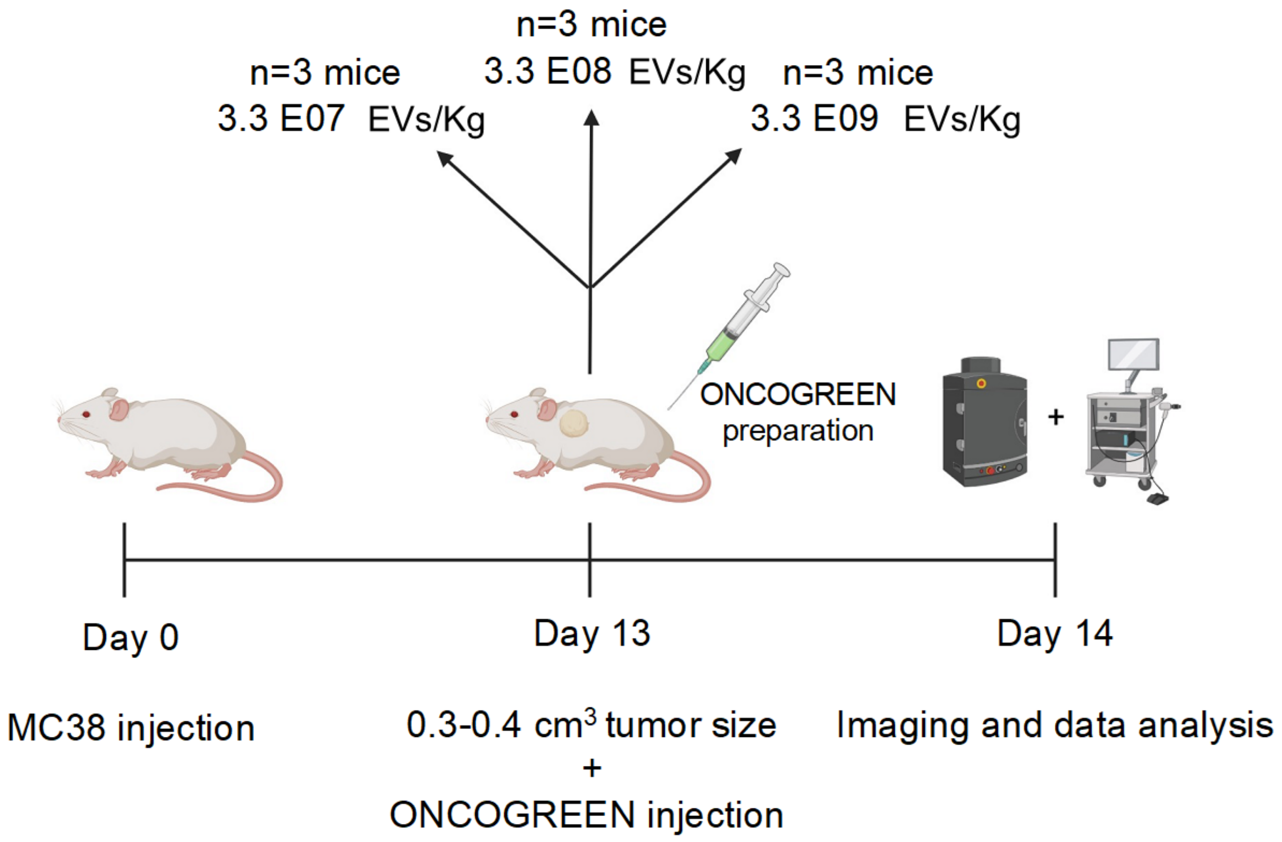

Supplementary Figure 2. Schematic representation of the experimental plan to determine the MED required for detecting tumor margins with intraoperative instrumentation. Three groups of C57BL/6 wild-type mice bearing tumors of 0.4 cm^3 sizes were intravenously treated with three different dosages of the ONCOGREEN formulation (3.3E07, 3.3E08, 3.3E09 EVs/Kg). In vivo and ex vivo images were acquired 24 hours post-injection using the IVIS Spectrum Imaging System and the SPY Elite intraoperative imaging device

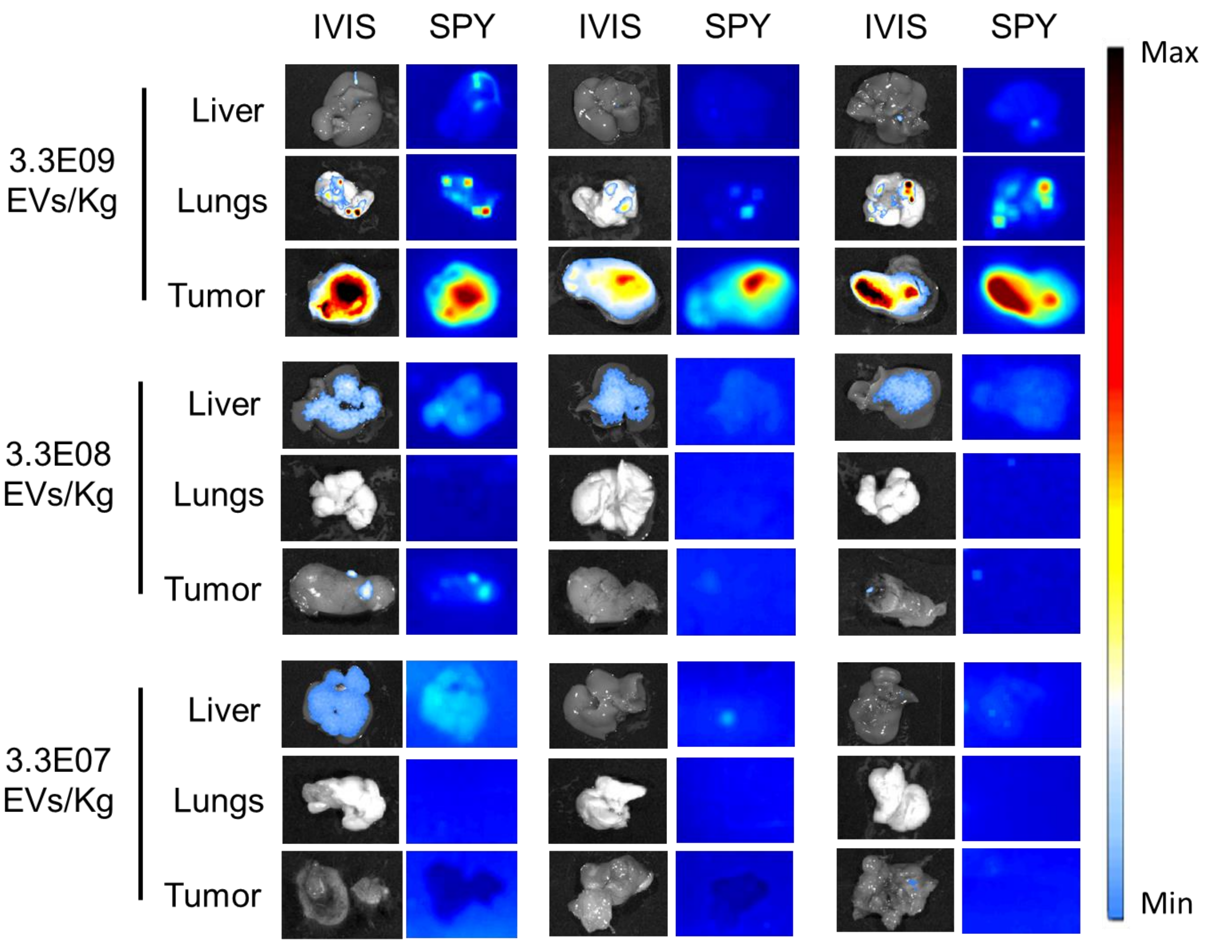

Supplementary Figure 3. Images acquired with the IVIS Spectrum Imaging System and SPY Elite intraoperative imaging device, depicting the fluorescence captured ex vivo in the liver, lungs, and tumor of two replicate mice treated intravenously with three dosages of nanoparticles – 3.3E09 EVs/Kg, 3.3E08 EVs/Kg, or 3.3E07 EVs/Kg for 24 hours. The color scale represents the fluorescence signal, with blue indicating the minimum intensity and red indicating the maximum.

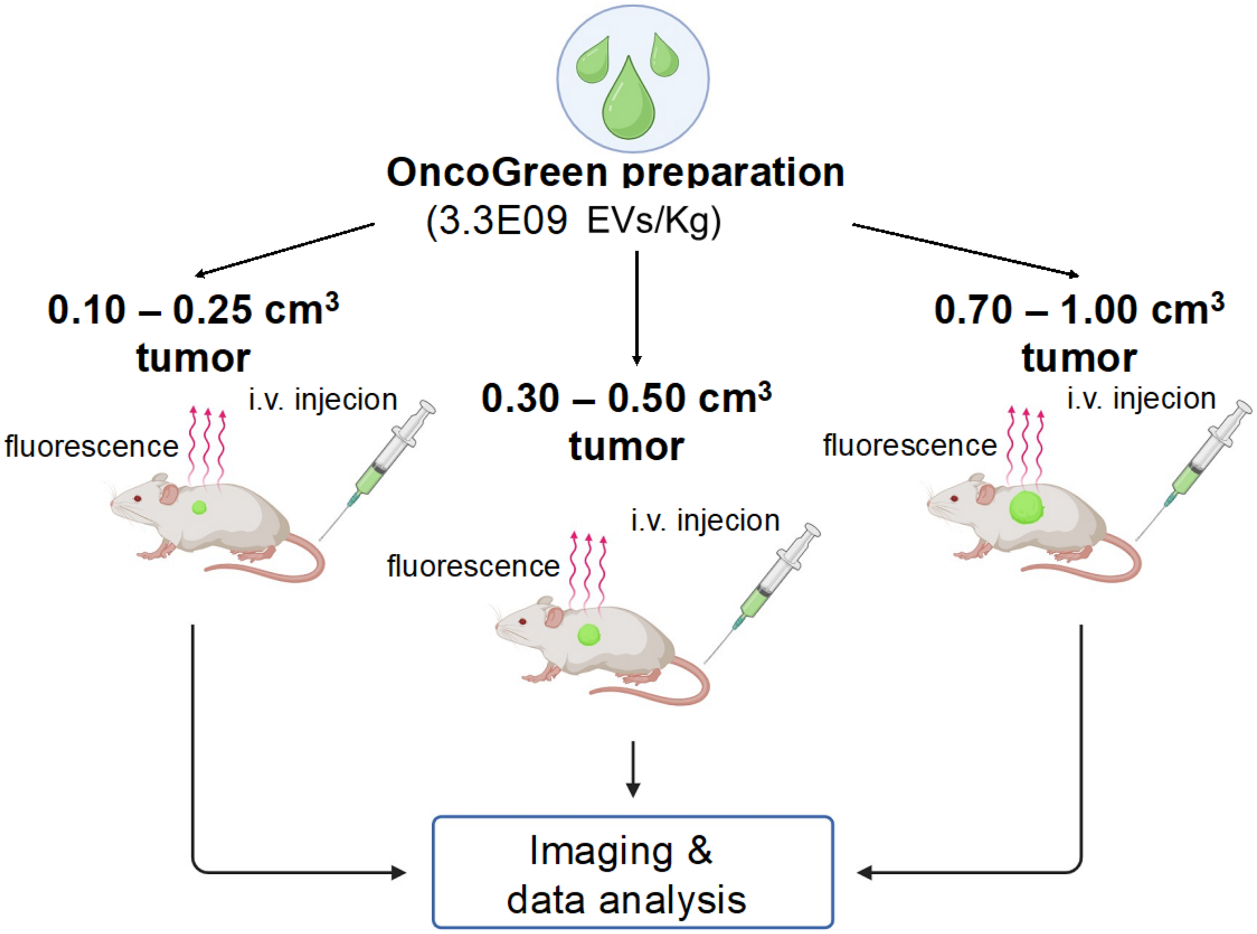

Supplementary Figure 4. Schematic representation of the experimental plan to test the effect of tumor size on the fluorescent signal accumulated in the neoplastic tissue. Three groups of mice bearing tumors of different sizes (small: 0.1-0.25 cm^3, medium: 0.3-0.5 cm^3, and large: 0.7-1.0 cm^3) were intravenously treated with 3.3E09 EVs/Kg of the ONCOGREEN formulation at the minimum effective dose (MED).

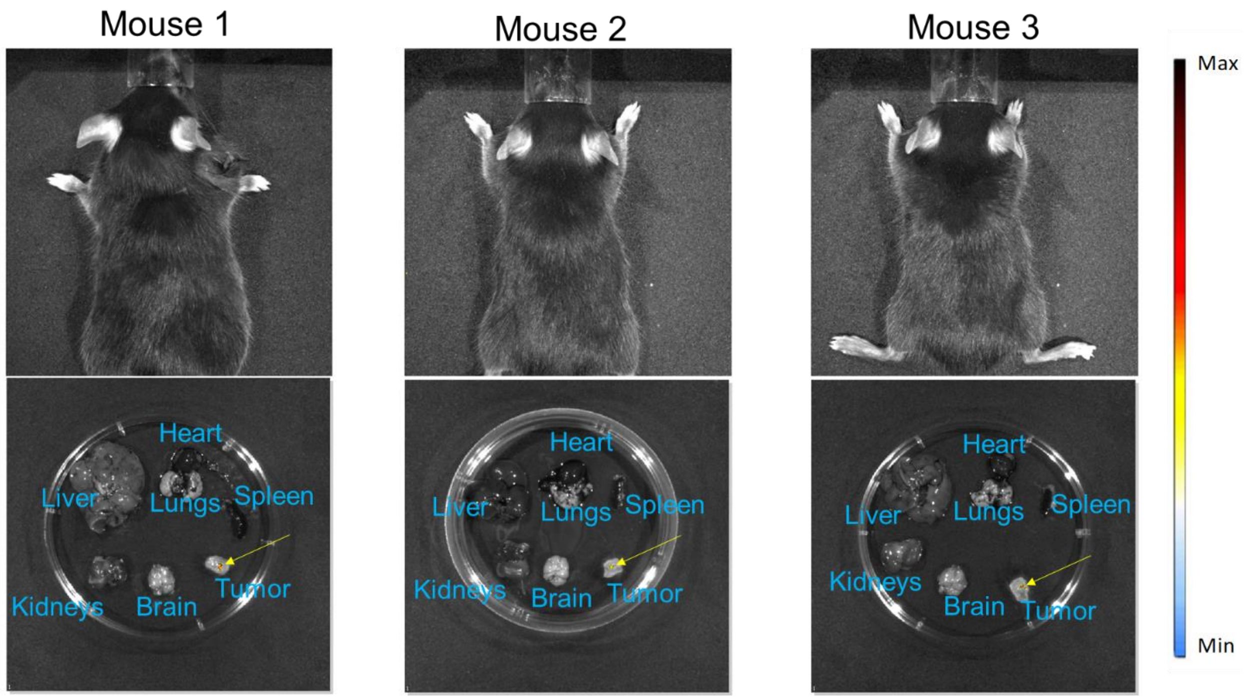

Supplementary Figure 5. mages acquired at 24 hours with the IVIS Spectrum Imaging System, depicting the *in vivo* (upper pictures) and *ex vivo* (lower pictures) biodistribution of PDEVs-ICG at 24 hours in n=3 mice bearing very small tumors (<0.1 cm^3). The tumors in the *ex vivo* pictures were collected together with surrounding healthy tissue: the tumor position was confirmed through macroscopic examination by a veterinary oncologist, and is indicated by the yellow arrow, and margins are highlighted by the green dotted line. The color scale represents the fluorescence signal, with black indicating the minimum intensity (10^7 p/sec/cm^2/sr) and yellow indicating the maximum (10^8 p/sec/cm^2/sr).

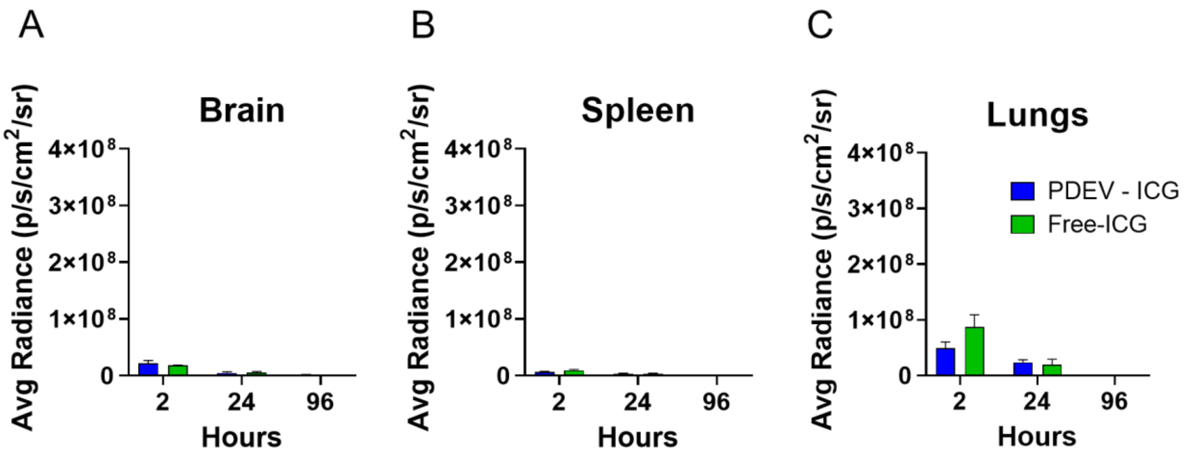

Supplementary Figure 6. The graphs depict the NIR fluorescence emission from additional mouse organs obtained from the experiment described in Figure 4.

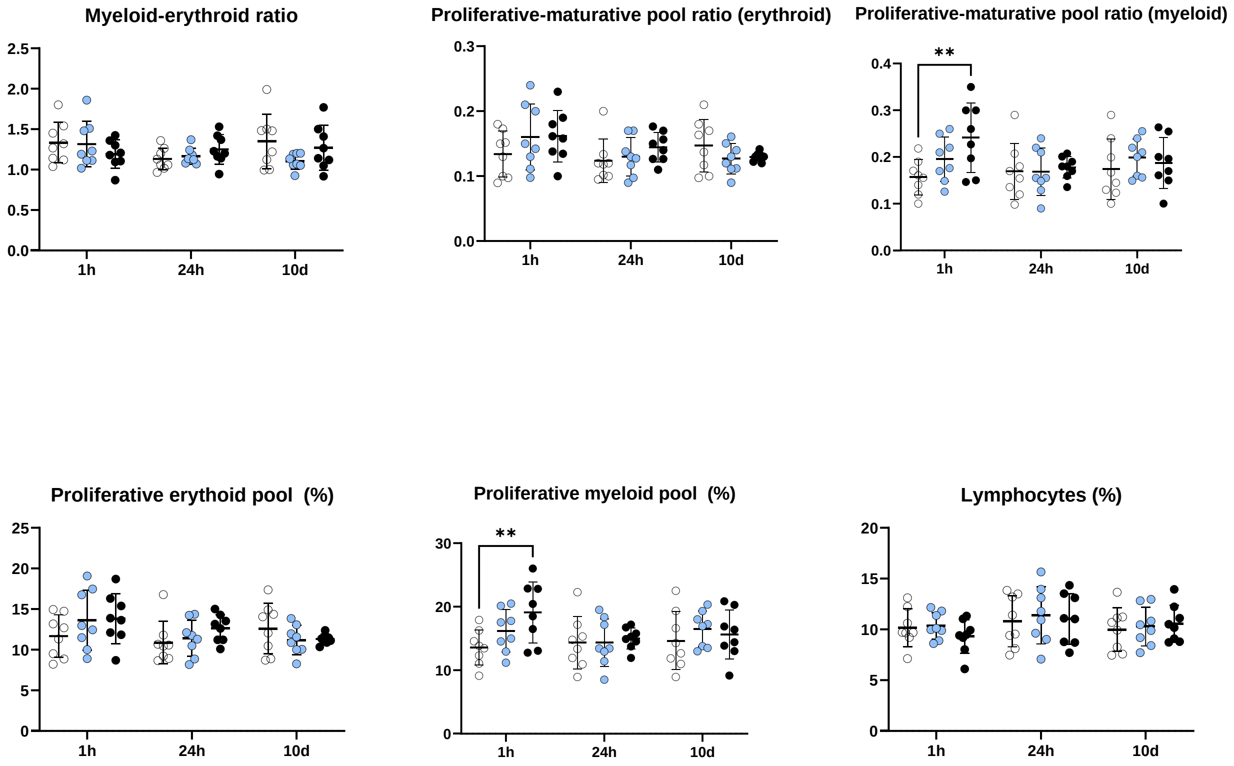

Supplementary Figure 7. Graphs representing bone marrow analyses on mice treated with vehicle (white dots), 3.3E09 EVs/Kg (cyan dots) and 3.3E10 (black dots), showing the values for the single animals.

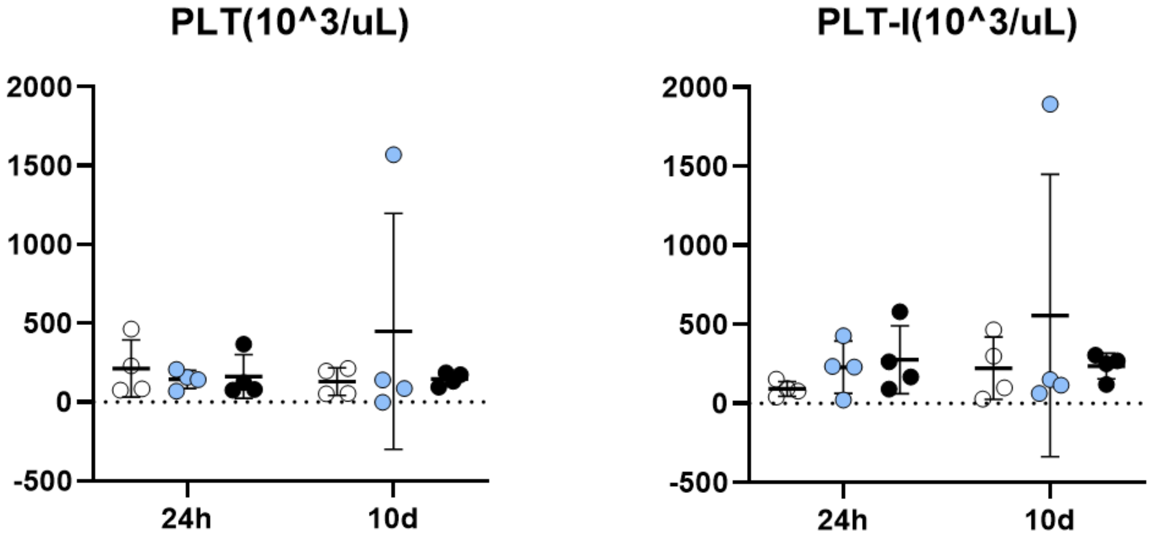

Supplementary Figure 8. Graphs representing platelet analyses (PLT: total platelet count; PLT-I: platelet impedance) on mice treated with vehicle (white dot), 3.3E09 EVs/Kg (cyan dot) and 3.3E10 (black dot), showing the values for the single animals.

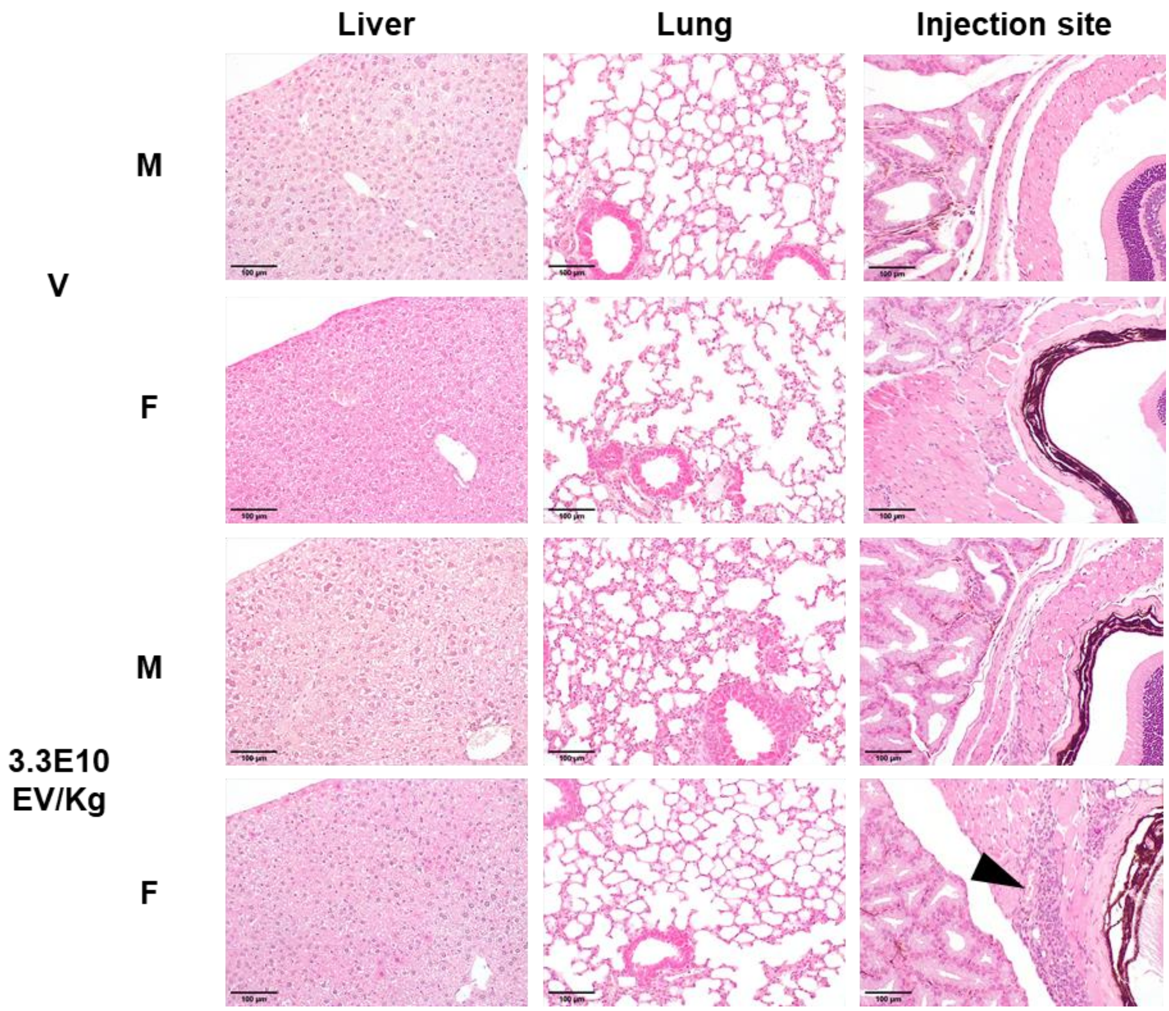

Supplementary Figure 9. Histology of liver, lung and injection site 10 days after the administration of Vehicle (V) and 3.3E10 EV/Kg (Hematoxylin & Eosin, 200x, scale bar = 100 μm). Microscopic changes were absent, except for a residual minimal macrophagic infiltration (arrowhead) in the injection site (retro-orbital region) observed in 1 female mice, suggesting a local injection-related effect undergoing recovery.

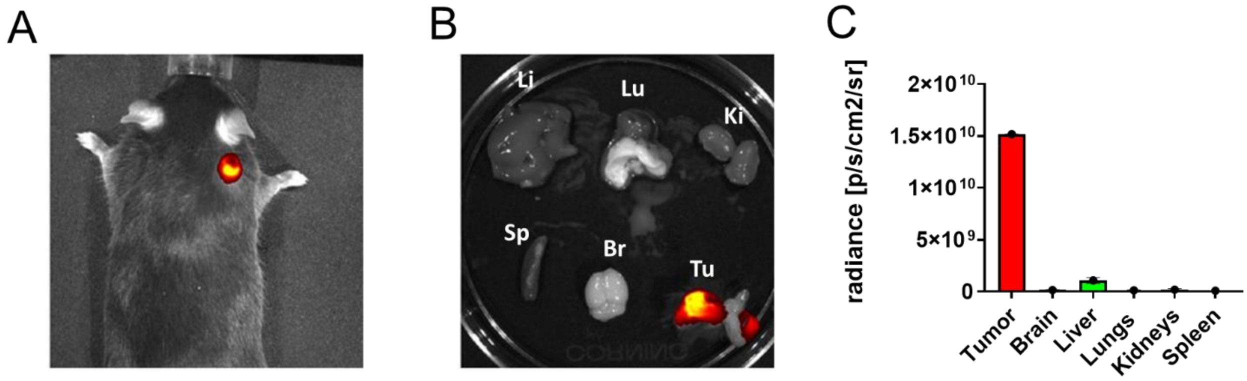

Supplementary Figure 10. Representative images acquired at 24 hours with the IVIS Spectrum Imaging System, depicting the fluorescence captured *in vivo* (A) and *ex vivo* (B) in the liver (Li), lungs (Lu), kidneys (Ki), spleen (Sp), brain (Br) and tumor (Tu) of three replicate mice treated intravenously with the MED of plasmapheresis-derived EVs loaded with ICG. The color scale represents the fluorescence signal, with black indicating the minimum intensity and yellow indicating the maximum. C) Quantification of the fluorescence signals.

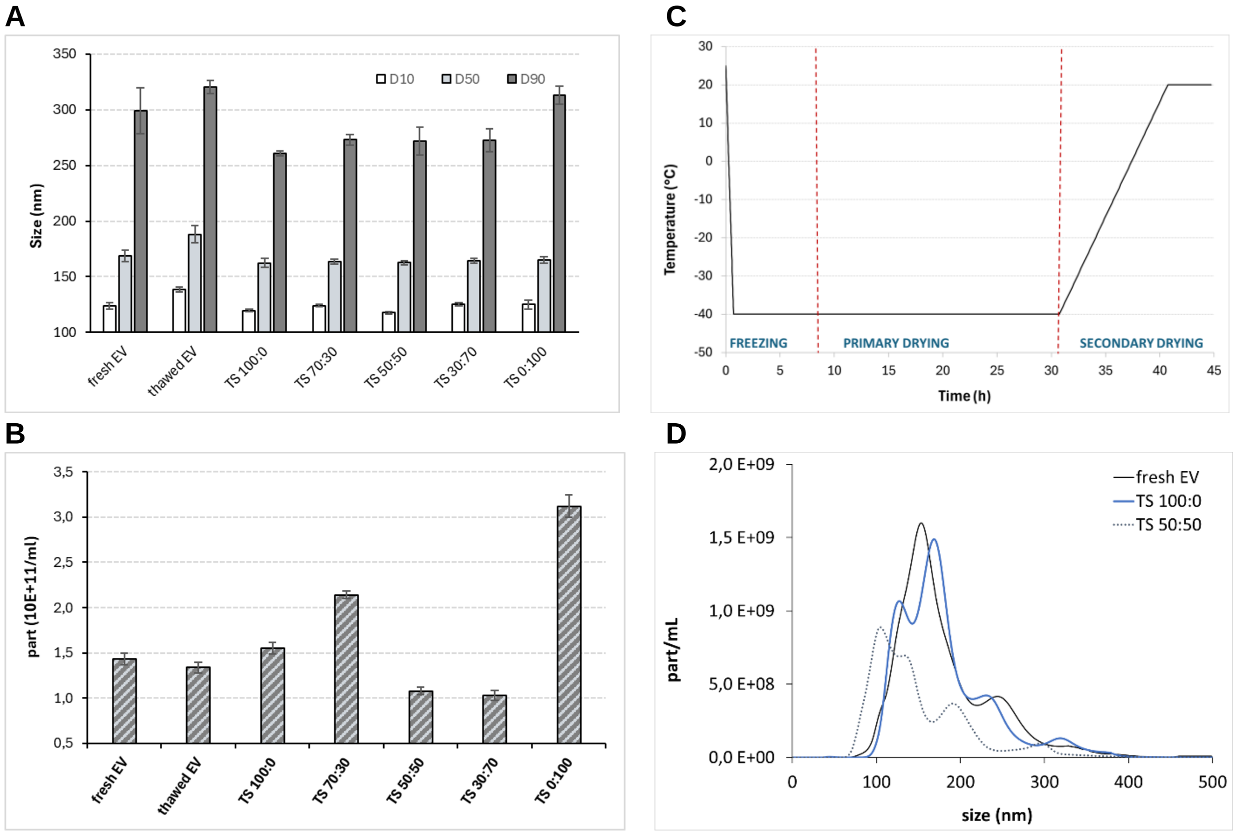

Supplementary Figure 11. The composition of medium in which EVs were dispersed was tuned up in order to satisfy three main requirements: (i) osmolarity ranging 285±15 mOsm/Kg, (ii) compatibility and (iii) cryo- and lyoprotection enabling the preservation of the bioactivity. Hence, EV dispersions in 10x PBS were mixed with a solution of trehalose or a trehalose:sucrose mixture (TS) in different ratios. The freeze thawing cycle caused a slight, but statistically significant, increase of EV dimensions (panel A) and a decrease of their concentrations (panel B). Among the tested excipients, only trehalose allowed to retain the size and the particle concentration with respect to the fresh EV, suggesting the possible use as cryoprotectant. The freeze-drying process, performed according to the process depicted in panel C, confirmed the suitability of trehalose in giving elegant cakes and preserving the physical features of EV (panel D).

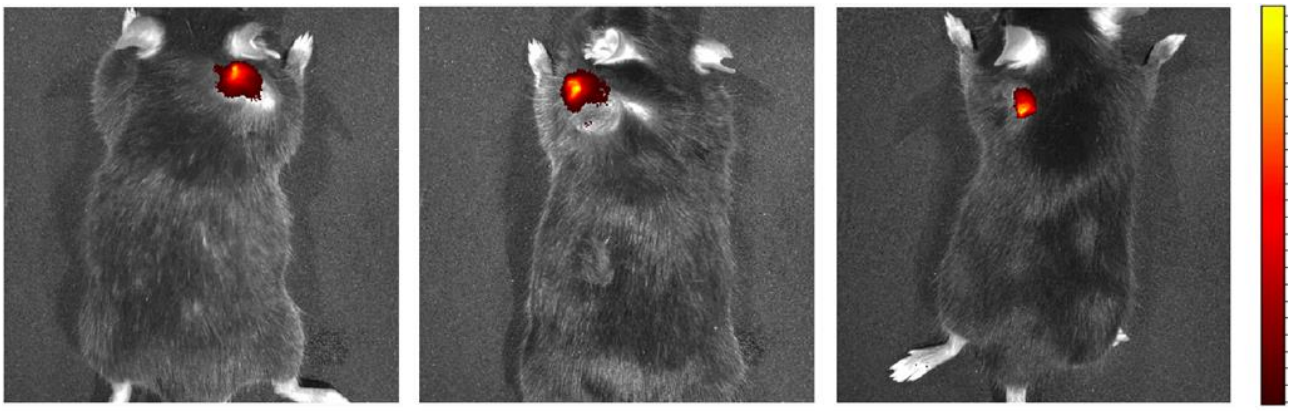

Supplementary Figure 12. Representative images acquired at 24 hours with the IVIS Spectrum Imaging System, depicting the *in vivo* biodistribution studies at 24 hours clearly demonstrating the retention of the tumor-targeting property in lyophilized (RT group) PDEVs. The color scale represents the fluorescence signal, with black indicating the minimum intensity (10^7 p/sec/cm^2/sr) and yellow indicating the maximum (10^8 p/sec/cm^2/sr).

		1h						24h						10d					
		V		3.3E09 EV/Kg		3.3E10 EV/Kg		V		3.3E09 EV/Kg		3.3E10 EV/Kg		V		3.3E09 EV/Kg		3.3E10 EV/Kg	
		M	F	M	F	M	F	M	F	M	F	M	F	M	F	M	F	M	F
	No. of examined mice/group	4	4	4	4	4	4	4	4	4	4	4	4	4	4	4	4	4	4
Liver																			
	thrombosis with hepatic necrosis, slight	0	0	0	0	0	0	0	0	0	0	1	0	0	0	0	0	0	0
Lung																			
	thrombosis	0	0	0	0	0	0	1	0	0	0	0	0	0	0	0	0	0	0
	arterial medial hypertrophy, slight	0	0	0	0	0	0	0	0	0	0	0	0	0	0	0	0	1	0
Injection site																			
	granulocytic infiltration	1	0	0	0	2	1	3	1	1	1	1	0	0	1	0	0	0	0
	macrophagic infiltration	0	0	0	0	0	0	0	0	0	0	2	2	0	0	0	0	0	1
	thrombosis	0	0	0	0	0	0	0	0	0	0	1	0	0	0	0	0	0	0

Supplementary Table 1. Prevalence of most relevant histopathological changes in mice treated with vehicle (V), 3.3E09 EVs/Kg and 3.3E10. The numbers refer to the number of animals showing the described deviation from physiology.

Parameter	Reference interval (Charles River)
Glucose (mg/dL)	172-372
Urea (mg/dL)	10-56
Creatinine (mg/dL)	0.2-0.5
Tot. Proteins (g/dL)	4.8-7.2
Albumin (g/dL)	2.4-4.3
ALT (U/L)	27-195
GLDH (U/L)	n.d.
ALP (U/L)	105-370
Chol (mg/dL)	55-169
CK (U/L)	n.d.
LDH (U/L)	n.d
Hemoglobin (g/dL)	10.8-19.2
Mean corpuscular hemoglobin (pg)	11.7-16.8
Mean corpuscular hemoglobin concentration (g/dL)	24.6-35.9
RBC (10^6/µL)	7.14-12.20
WBC (10^3/µL)	3.90-13.96
Neutrophils%	7.36-28.59
Eosinophils%	0.13-4.51
Basophils%	0.01-1.26
Lymphocytes%	61.26-87.82
Monocytes%	2.18-11.02
Platelets (10^3/µL)	565-2159

Supplementary Table 2. Reference intervals (released by Charles River for the C57BL/6 mouse strain) for specific parameters investigated in the study and reported in Table 3 and Table 4.
